# Crystal structures of mono- and bi-specific diabodies and reduction of their structural flexibility by introduction of disulfide bridges at the Fv interface

**DOI:** 10.1038/srep34515

**Published:** 2016-09-29

**Authors:** Jin Hong Kim, Dong Hyun Song, Suk-Jun Youn, Ji Won Kim, Geunyoung Cho, Sun Chang Kim, Hayyoung Lee, Mi Sun Jin, Jie-Oh Lee

**Affiliations:** 1 Graduate School of Nanoscience and Technology, KAIST, Daejeon, Korea; 2 Agency for Defense Development, Daejeon, Korea; 3 Department of Chemistry, KAIST, Daejeon, Korea; 4 Department of Biological Sciences, KAIST, Daejeon, Korea; 5 Institute of Biotechnology, Chungnam National University, Daejeon, Korea; 6 School of Life Sciences, GIST, Gwangju, Korea

## Abstract

Building a sophisticated protein nano-assembly requires a method for linking protein components in a predictable and stable structure. Diabodies are engineered antibody fragments that are composed of two Fv domains connected by short peptide linkers. They are attractive candidates for mediators in assembling protein nano-structures because they can simultaneously bind to two different proteins and are rigid enough to be crystallized. However, comparison of previous crystal structures demonstrates that there is substantial structural diversity in the Fv interface region of diabodies and, therefore, reliable prediction of its structure is not trivial. Here, we present the crystal structures of ten mono- and bi-specific diabodies. We found that changing an arginine residue in the Fv interface to threonine greatly reduced the structural diversity of diabodies. We also found that one of the bispecific diabodies underwent an unexpected process of chain swapping yielding a non-functional monospecific diabody. In order to further reduce structural flexibility and prevent chain shuffling, we introduced disulfide bridges in the Fv interface regions. The disulfide-bridged diabodies have rigid and predictable structures and may have applications in crystallizing proteins, analyzing cryo-electron microscopic images and building protein nano-assemblies.

Linking protein components to form a predictable and rigid structure is a prerequisite for generating complex protein assemblies in a predetermined fashion. Most of the chemical cross linkers available have long and flexible spacers. Because of this the linked proteins have significant structural flexibility, and the relative orientation and distance between their protein components is largely unpredictable. This is the case even when the chemical cross linkers themselves have rigid structures since they are attached to flexible side chains such as cysteines or lysines. Recently several research groups have reported novel methods that can produce artificial protein assemblies with predictable and stable structures^1^,^2^,^3^,^4^,^5^. However, the application of these methods to a wide range of protein structures requires more research.

Recombinant bispecific antibodies are artificial proteins containing the antigen- binding domains of two different antibodies^6^. They are being actively studied because their “two-target” functionality can improve the therapeutic value of the individual antibodies by causing hetero-dimerization of antigens. Currently more than 50 different recombinant formats are being developed for generating bispecific antibodies and many of the latter are in various stages of clinical trial against cancers and inflammatory diseases. Diabodies are among the smallest of these bispecific antibodies. They are constructed by connecting heavy-chain variable fragments (V_H_) and light-chain variable fragments (V_L_) with short “GGGGS” peptide linkers^7^. The five-amino-acid linker is too short to span the distance of ~35 angstroms from the V_H_ C terminus to the V_L_ N terminus. Therefore, the two V_H_-linker-V_L_ chains associate to form a dimer with two antigen binding sites, each consisting of the V_H_ and V_L_ fragments from the different chains.

When the two Fv domains in the diabody are identical, the structure is called a monospecific diabody. Such diabodies induce homo-dimerization of target proteins because they can simultaneously bind two identical protein molecules. On the other hand, bispecific diabodies have Fv domains originating from two different antibodies and so can bridge different proteins leading to their hetero-dimerization. They can be used to connect a wide range of molecules because antibodies can be generated against almost any kind of molecule. Hundreds of thousands of antibody clones are developed each year. Furthermore, more than 2,000 antibody structures with and without bound antigen have already been deposited in the Protein Data Bank (PDB). Diabodies can easily be produced from this huge array of antibodies and can be used as building blocks to construct artificial protein assemblies for a variety of applications.

The crystal structures of several monospecific diabodies have been reported^8^,^9^. In these structures, the two Fv domains of the diabodies are arranged in tail-to-tail fashion. Their antigen binding sites point in roughly opposite directions and the structure is rigid enough to be crystallized. On the other hand, antigen binding sites in intact antibodies or in most other bispecific antibody constructs are connected by flexible hinge structures, and their relative orientations are not fixed. In the crystal structures of diabodies, the light chains do not contribute to the interaction between the Fv domains and the two heavy chains form a relatively small interaction interface. Recently reported structure of a diabody against CD47 is an exception^10^. In this diabody, both light and heavy chains contribute to the extensive interaction interface. Generally, the variable domains of the heavy, V_H_, and light chains, V_L_, are genetically fused in the order V_H_-V_L_. However, Carmichael *et al*. have reported the structure of an unusual diabody in which V_L_ precedes V_H_^11^. This structure has a different arrangement of the Fv domains, demonstrating the structural diversity of diabodies.

In order to use diabodies as general and reliable mediators of artificial protein assemblies, one must be able to predict the orientation and distance between the two bound proteins with great accuracy. Therefore, the structures of diabodies must be rigid and predictable. The individual Fv domains of diabodies do have rigid and predictable structures as shown in the thousands of antibody crystal structures deposited in the PDB. However, the interfaces between the two Fv domains in the diabodies differ substantially in sequence and appear to be too small to have stable structures^8^,^9^. In order to investigate the structural flexibility of the Fv interfaces of diabodies, we have determined the crystal structures of 10 mono- and bi-specific diabodies with and without bound antigens. These structures demonstrate that diabodies are readily crystallizable but that, in the current format, the interfaces between the two Fv domains are too diverse and it is difficult to accurately predict their structures. We show that substitution of an arginine residue in the EF loop and introduction of a disulfide bridge between the Fv domains renders the diabody structure rigid and more predictable.

## Results

### Structural diversity of diabodies

**Figure 1:**
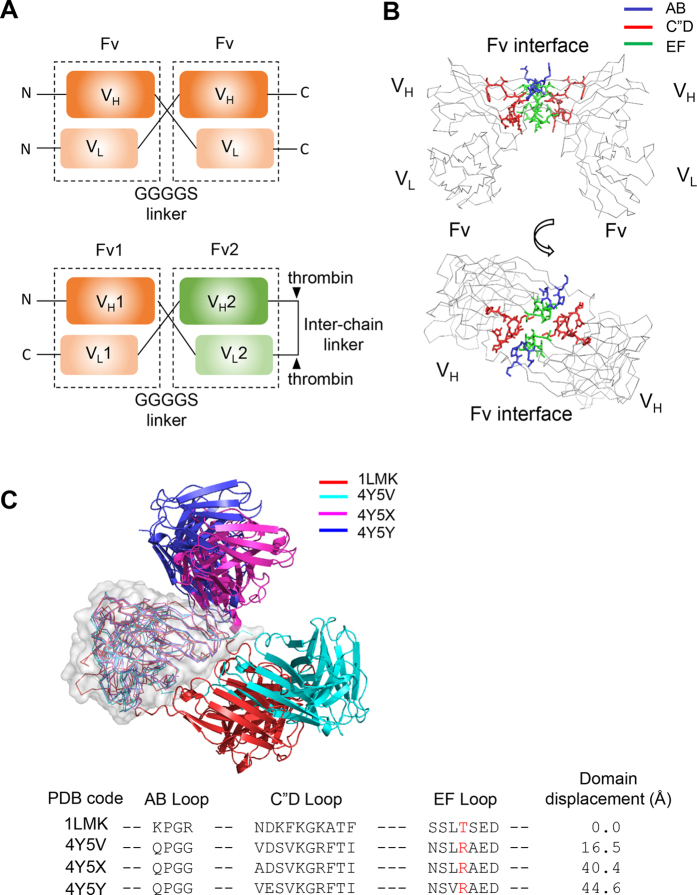
Structural comparison of monospecific diabodies. (**A**) Schematic diagram of mono- and bi-specific diabodies. (**B**) Cα trace of monospecific diabody with the PDB code, 1LMK^9^. The AB, C”D and EF loops critical for formation of the Fv interface are colored in blue, red and green, respectively. (**C**) Structural comparison of the previously reported structures of four monospecific diabodies^8^,^9^. The amino acid sequences of the AB, C”D and EF loops are written beneath the figure. The amino acid in position 83 according to the Kabat numbering scheme^40^ is colored in red. To measure “domain displacement”, one of the Fv domains of the reference and the target diabodies were structurally aligned and the distances between the Cα carbons of the Trp36 residues in the heavy chains of the second Fv domains were measured. The Trp36 residues are located in the hydrophobic core of the Fv domains and their structures are practically invariant in all antibody structures. The 1LMK diabody was chosen as reference molecule for this calculation.

In monospecific diabodies, two identical V_H_-V_L_ protein chains form a homo dimer (Fig. 1A). To generate bispecific diabodies, two non-identical V_H_-V_L_ chains split by thrombin form a heterodimeric complex. In order to understand the structural diversity of diabodies, we inspected the previously reported crystal structures of a monospecific diabody with the PDB code, 1LMK^9^. In this structure the Fv interface is composed mainly of the AB, C”D and EF loops of the heavy chains, and the light chains are not involved in the interaction between the Fv domains (Fig. 1B and Supplementary Fig. S1). The surface area of the Fv interface is relatively small and contains no hydrophobic residues or hydrogen bond networks. This suggests that the Fv interface is not stable and could be perturbed by small changes in the amino acid sequences. Recently, the structures of three monospecific diabodies containing different amino acid sequences in the C”D and EF loops have been reported^8^. As expected, their structures show large displacements of the second Fv domains after alignment of one of the Fv domains (Fig. 1C). In fact the Fv domains of diabodies 4Y5X and 4Y5Y are displaced by more than 40 angstroms relative to that of 1LMK. The A, C, G and F strands, instead of the C”D and EF loops, play the main role in this new Fv interface. The large structural difference between diabodies demonstrates that prediction of their structures is not trivial and would limit their use in the assembly of protein nano-complexes.

In order to understand why the reported structures of the four diabodies have such large domain displacements, we closely inspected the amino acid sequences in the Fv interfaces (Fig. 1C). Several amino acids in the C”D and EF loops showed significant sequence variation. The amino acid in position 83 of the EF loop is located in the center of the Fv interface and appears to play a critical role in determining the structure of the interface. Arginine and threonine are the most common amino acids found in this position. In the 1LMK structure, the distance between the hydroxyl oxygens of the two Thr83 side chains is only 8 angstrom^9^. In the other three diabodies, this position is occupied by an arginine, which has a long and positively charged side chain. Therefore, in these diabody structures the two Arg83 residues should strongly repel each other and push the Fv domains apart. Indeed, the guanidinium groups of the arginine side chains are separated by 20, 30 and 36 angstroms in the crystal structures of diabodies 4Y5V, 4Y5X and 4Y5Y, respectively. This analysis suggests that arginine in position 83 makes the structure of the Fv interface more ambiguous and unpredictable. In order to confirm this, we determined the structures of six additional monospecific- and bi-specific diabodies with amino acid substitutions at position 83.

### Structures of mono- and bi-specific diabodies containing Thr83 in the EF loop

**Figure 2:**
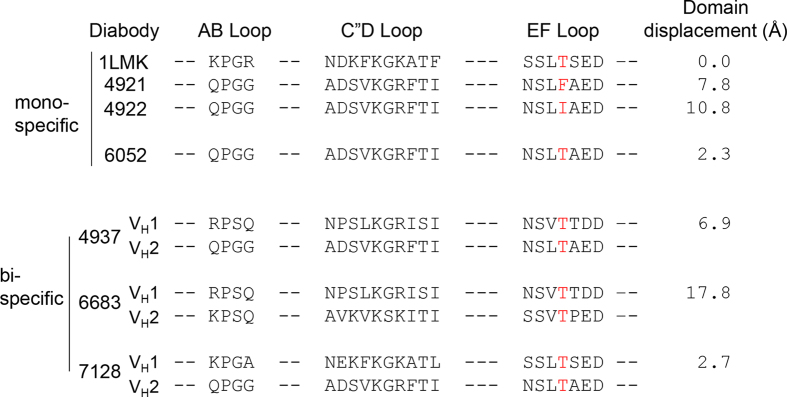
Substitution of Arg83 with a threonine in the EF loop of diabody. Amino acid sequences of the AB, C”D and EF loops are shown. “Domain displacements” are calculated as in Fig. 1C.

**Figure 3:**
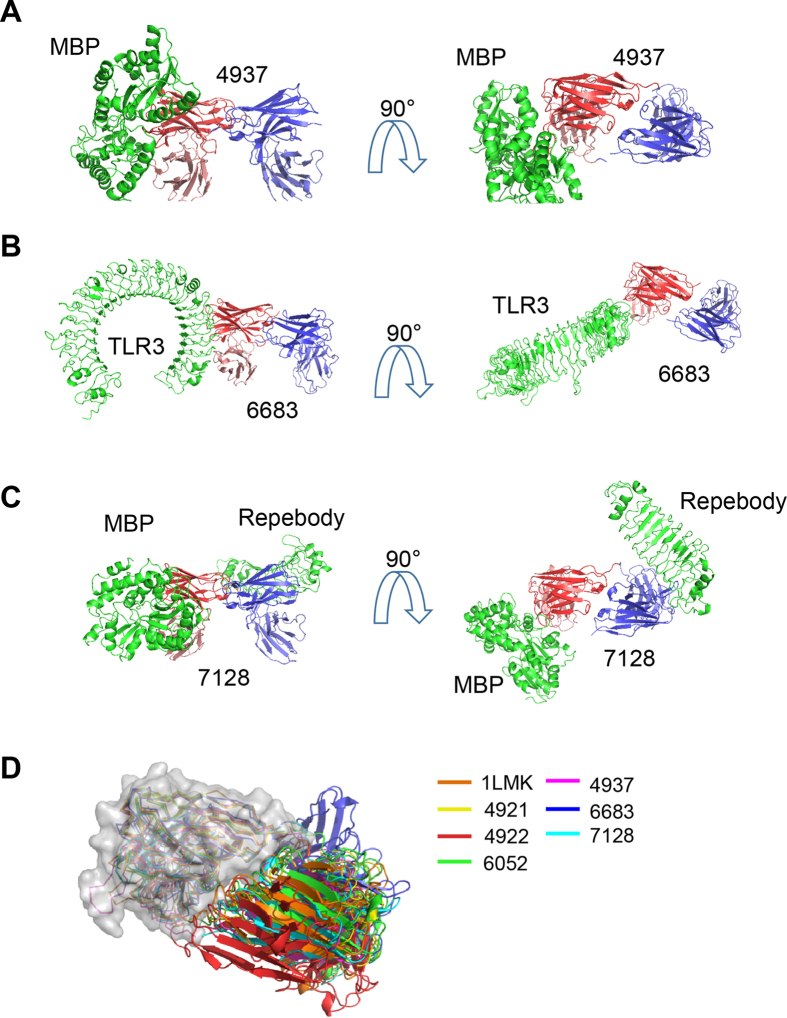
Crystal structures of the bispecific diabody-antigen complexes. (**A**) Crystal structure of the MBP-49347 complex. (**B**) Crystal structure of the TLR3-6683 complex. (**C**) Crystal structure of MBP-7128-Repebody complex. (**D**) Superimposition of diabody structures. The second Fv domains are drawn schematically after structural alignment of one of the Fv domains of the diabodies.

We determined the structures of three monospecific and three bispecific diabodies by substituting the arginine at position 83 with other amino acids (Fig. 2). Note that the diabodies we used had substantial sequential diversity in their C”D and EF loops. The bispecific diabodies were produced in single chain format by inserting a 42 amino acid linker between the two V_H_-V_L_ chains (Fig. 1A and Supplementary Fig. S2)^12^,^13^,^14^,^15^. This highly flexible linker is harmful for crystallization and was removed by thrombin cleavage after the protein had been produced. The structures of the bispecific diabodies were determined in complex with bound protein ligands (Fig. 3A–C). These are the first crystal structures of bispecific diabodies.

In order to evaluate the role of Arg83 in the structural diversity of the Fv interface, we compared the structures of the six diabodies with that of 1LMK by aligning one of the Fv domains. As shown in Fig. 3D, the second Fv domains of all the diabodies are clustered in a small area, demonstrating that substitution of the positively charged arginine greatly reduces the structural diversity of diabodies. However, as shown in Fig. 2, the “domain displacements” of monospecific diabody 4922 and bispecific diabody 6683, are of more than 10 angstrom demonstrating that diversity of the structure of the Fv interface, although significantly reduced, is still substantial.

### Chain shuffling of a bispecific diabody

**Figure 4:**
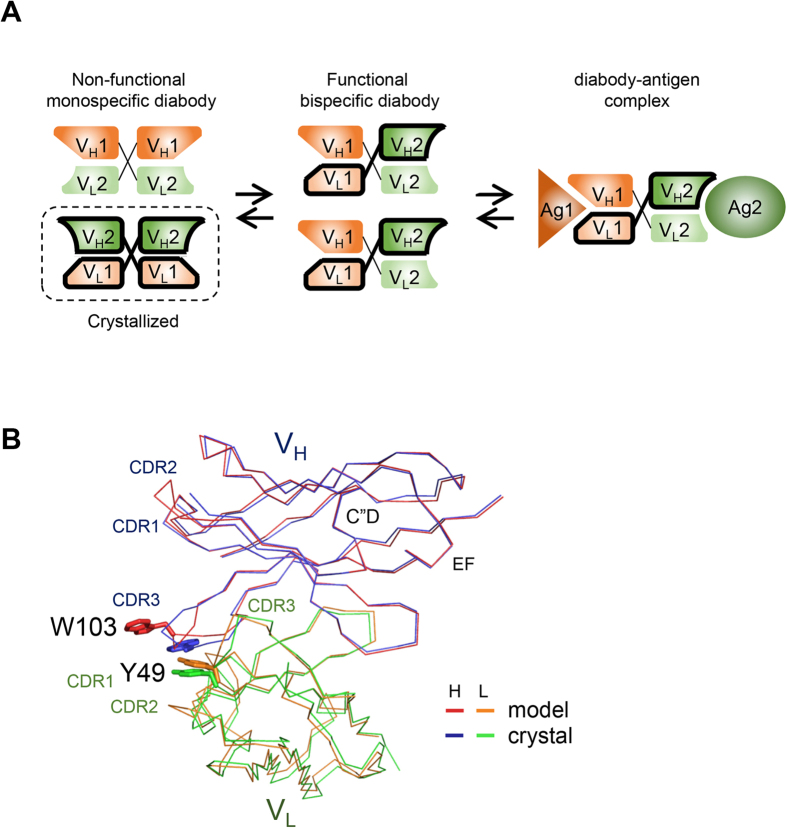
Swapping of the chains in bispecific diabodies. (**A**) The two chains in some bispecific diabodies can dissociate and form nonfunctional monospecific diabodies. (**B**) Comparison of the crystal structure of the chain-swapped monospecific diabody 6277 and a model of the intended functional bispecific diabody.

During this study, we by accident determined the structure of a non-functional monospecific diabody. We had intended to produce a bispecific diabody, 6277, and to try to determine its structure. To our surprise, instead of forming a functional bispecific diabody as expected, the two chains of the diabody had been shuffled and a non-functional monospecific diabody had been crystallized (Fig. 4A and Supplementary Fig. S3). Before thrombin cleavage, the single chain diabody was monomeric and functional, as shown previously^12^,^13^,^14^,^15^ and in Supplementary Figs S4 and S5. This suggests that cleavage and removal of the linker is responsible for chain shuffling.

In order to understand what caused the chain shuffling, we compared the crystal structure of the shuffled monospecific diabody and predicted model of the intended bispecific diabody (Fig. 4B). The predicted model was extracted, without modification, from the Fv part of a published structure with PDB code number 2V7N^16^. The core part of the interface between the heavy and light chains is conserved in both the shuffled and the modeled Fv domains. The main difference in the structures is found in the CDR3 loop of the heavy chain: in the crystal structure of the shuffled diabody, this loop is displaced by ~4.5 angstrom relative to that of the modelled bispecific diabody. This movement appears to have been triggered by interaction between Trp103 of the CDR3 loop of the heavy chain and the Tyr49 of the CDR2 loop of the light chain. However, it seems that this interaction is not strong enough to freeze the structure into the non-functional form because the purified diabody is actually functional and can bind to antigens TLR3 and CitS. Therefore, it seems likely that in solution our diabody consists of functional bi- and nonfunctional mono-specific diabody molecules in equilibrium (Fig. 4A), and the presence of an antigen “forces” the molecules into the functional bispecific form. However, the nonfunctional monospecific diabody form appears to be selectively crystallized under our crystallization conditions.

### Design of disulfide bridges in the Fv interface

**Figure 5:**
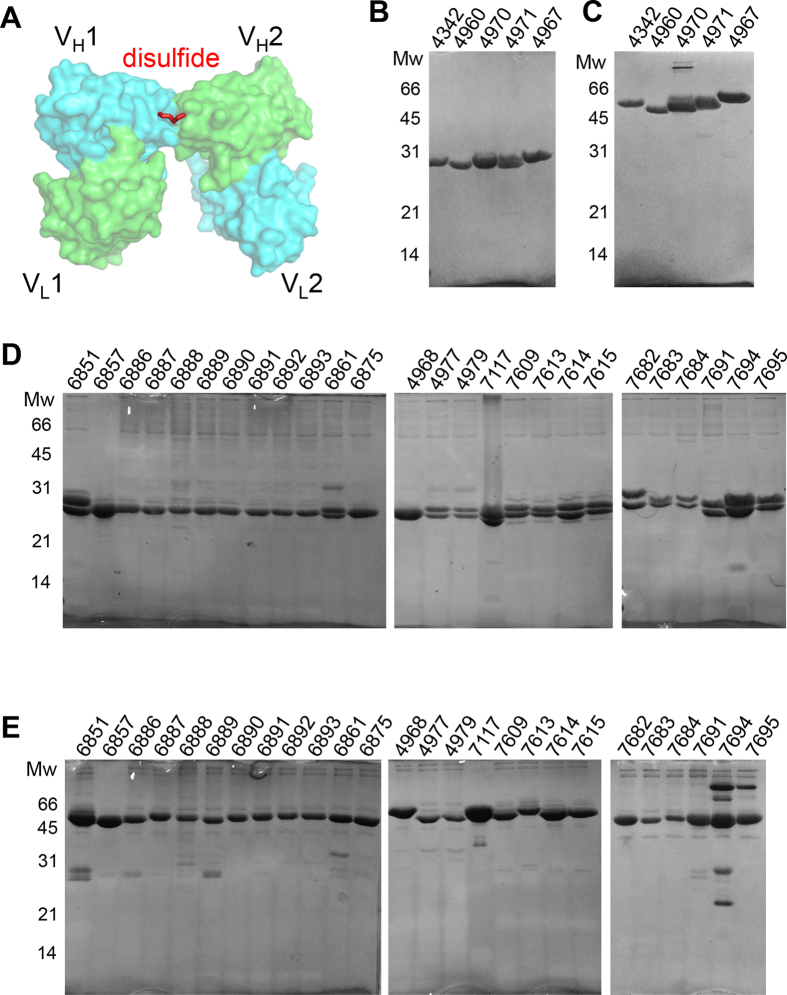
Formation of disulfide bridges between the Fv domains in mono- and bi-specific diabodies. (**A**) Disulfide bridges were introduced between the heavy chains of the Fv interface. The amino acid sequences of the mutated diabodies are shown in Fig. 5 and Supplementary Figures S6–15. The formation of disulfide bridges was confirmed by comparing reducing and non-reducing SDS-PAGE analyses. For the reducing SDS-PAGE (**B**) and (**D**), 100 mM DTT was added to the sample buffer. The two bands correspond to the heavy and light chains of the Fv domains. Some of the diabodies appear to have only a single band because the heavy and light chains have similar molecular weights. In non-reducing SDS-PAGE (**C**) and (**E**), DTT was omitted from the sample buffer. The gels were stained with Coomassie Brilliant Blue.

**Figure 6:**
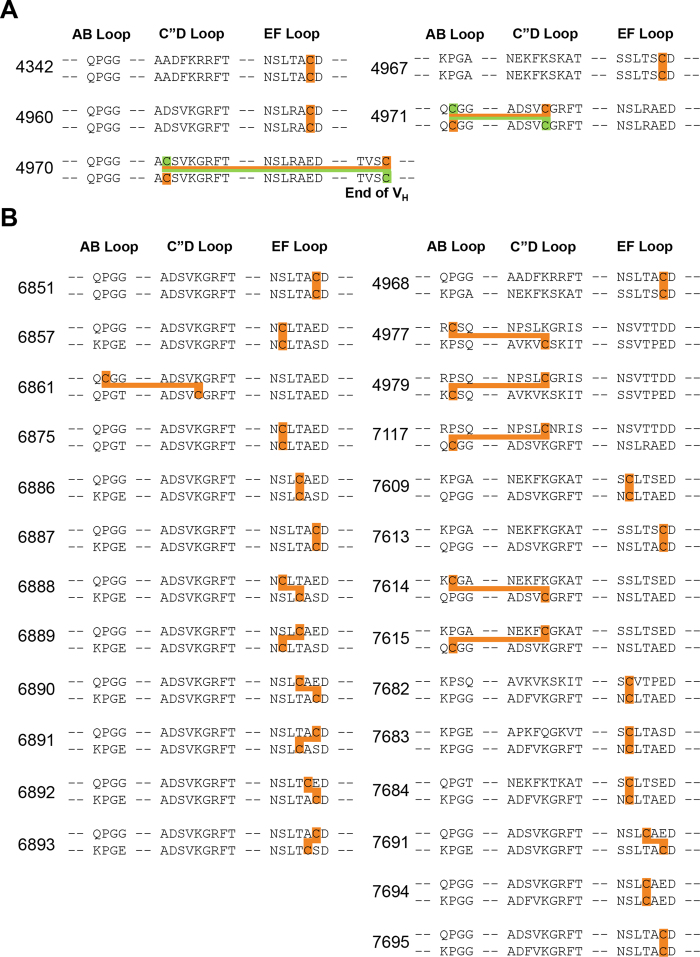
Design of the disulfide bridged diabodies. (**A**) monospecific diabodies. The cysteines connected by the disulfide bridges are marked with orange and green bars. (**B**) bispecific diabodies.

Although changing Arg83 to a threonine substantially reduces ambiguity in predicting diabody structures, they remain significantly diverse, which limits their use in designing protein nano-assemblies. Furthermore, chain shuffling of bispecific diabodies can substantially reduce binding, if not totally prevent it. In order to reduce this structural flexibility and prevent chain shuffling, we introduced disulfide bridges between the V_H_1 and V_H_2 chains in the Fv interface (Fig. 5A). The sites of the proposed cysteines were designed initially using the “Disulfide by Design” program, with the crystal structure of diabody 6052 as template (Supplementary Fig. S6)^17^. Later, we found that structure-based design was not essential and most of the exposed amino acids in the C”D or EF loops could be used for disulfide bridge formation with a high success rate (Figs 5B–E and 6 and Supplementary Figs S7–16). This is presumably due to the structural flexibility of the Fv interface. Among the 36 mutations we introduced, 31 were successfully produced in insect cells using the baculovirus expression system (Fig. 5B–E). Formation of the disulfide bridge was indicated by a band shift on non-reducing SDS-PAGE because the disulfide bridge increases the molecular weight of the diabody two-fold in non-reducing conditions. All the diabodies that could be produced in insect cells were up-shifted in non-reducing SDS-PAGE, confirming successful formation of the disulfide bridge. Two of the diabodies, 7694 and 7695, contained higher molecular weight complexes on the non-reducing gels, suggesting that tetrabodies had been formed by combining pairs of diabodies^18^,^19^. The formation of disulfide bridges appeared to be incomplete in the case of some of the diabodies, most notably 6851 and 6889.

**Figure 7:**
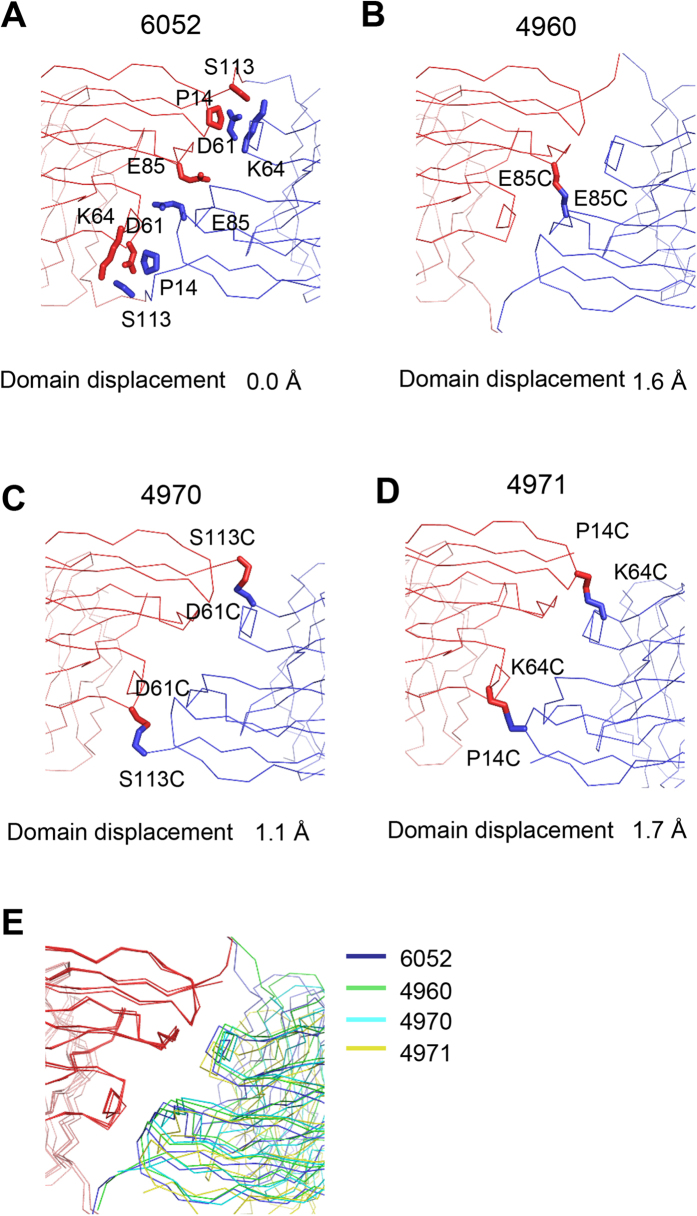
Structures of disulfide-bridged diabodies. The names of the diabodies are written above the figures. The two Fv domains are colored red and blue. The Cα structures of wild type diabody 6052 (**A**) and its E85C (**B**), D61C-S113C (**C**), P14C-K64C (**D**) mutant derivatives are shown. The cysteine side chains forming the disulfide bridges are shown. The “domain displacements” were calculated as in Fig. 1C, using the structure of the wild type 6052 as reference. (**E**) Superposition of the wild type and mutant structures.

In order to examine the structural flexibility of the disulfide-bridged diabodies, we determined the structures of three of them (Fig. 7). All were designed by the “Disulfide by Design” program using the crystal structure of the 6052 diabody as template (Fig. 7A and Supplementary Fig. S6)^17^. As shown in Fig. 7E, the three diabodies have practically identical structures regardless of large variations in the crystallization conditions (Supplementary Table S1). Some of them were crystallized in a buffer containing a high concentration of salt and organic solvent while others were generated in a buffer containing polyethyleneglycol. The pH of the crystallization solution ranged from 3.5 to 8.5. This demonstrates that the introduction of a disulfide bridge can essentially freeze the structure of a diabody under a wide range of experimental conditions.

In conclusion, we have found that substitution of the positively charged arginine in the EF loop greatly reduces the ambiguity in predicting diabody structure. The structural diversity is further reduced by introducing a disulfide bridge in the Fv interface. The structures of disulfide-bridged diabodies are rigid and should be predictable with high accuracy once we have a sufficient number of structures of the major diabody classes.

## Discussion

We have studied the structural diversity of diabodies by determining the crystal structures of several mono- and bi-specific examples and their complexes with antigens. We found that the Fv domain interfaces of the diabodies are structurally diverse because the interaction interface between the two Fv domains is small and lacks hydrophobic residues. We also found that the two chains of diabodies can dissociate and re-equilibrate. Therefore a non-functional combination of the heavy and light chains can predominate in some of the diabody populations. This could reduce the binding affinity of a diabody, and in extreme cases might totally abolish binding. Although this possibility has been proposed previously^12^,^20^,^21^,^22^,^23^, ours is the first structural evidence confirming the idea.

We introduced disulfide bridges in order to prevent chain shuffling and enhance the structural rigidity and predictability of diabodies. Because disulfide bridges link the two chains covalently, they can also block unwanted chain shuffling (Fig. 5A). Rigidity and predictability of structure are crucial when diabodies are used in designing complex protein assemblies. It has been previously reported that a disulfide bridge between the heavy and light chain inside the Fv domain can improve the stability of the diabody *in vitro* well as *in vivo*^24^. However, the disulfide bridges that were introduced were located within one Fv domain, not between Fv domains. Therefore, although such disulfide bridges can make the diabodies more stable, they cannot increase the rigidity or predictability of the relative conformation of the two Fv domains. The disulfide bridges we introduced were located between the Fv domains and can essentially lock-in the relative conformation of the Fv domains in the diabody.

Recently, Moraga *et al*. have shown that diabodies can be used as modulatory agents in receptor signaling^8^. They generated several monospecific diabodies against the erythropoietin receptor and found that some had an agonistic effect, presumably due to dimerizing the receptor. They also found that it was possible to vary the agonistic effect of a diabody by changing the relative distances and orientations of the two receptor molecules bound to the diabody. The diabodies they used had arginines in position 83 and therefore their Fv interfaces should have had significant structural flexibility. Here, we have shown that one can stabilize the relative orientation and separation of the bound proteins by introducing disulfide bridges. Furthermore, by changing the position of these disulfide bridge, it should be possible to control the relative orientation and distance of the bound target proteins. Therefore, our method might be of use in fine-tuning the agonistic activity of diabodies by altering the position of disulfides.

Diabodies stabilized by a disulfide bridge in the Fv interface could have a variety of uses. Many proteins and protein complexes, including transmembrane or large protein complexes, are notoriously difficult to crystallize. Antibody Fab domains have proved useful in crystallizing such challenging proteins by providing a new crystallization surface or hiding regions harmful for crystallization^25^. However, because the effect of the Fab domains on crystallization is difficult to predict, one often needs to screen many antibody clones to find the best one for crystallization. Because the production of a monoclonal antibody takes a long time and large resources, Fab domains are not widely used in routine crystallography. Diabodies, particularly those stabilized by an interface disulfide bridge, can be more useful for protein crystallization than simple Fab domains for the following reasons. First, they have all the advantage possessed by Fab domains; they can hide harmful surface areas and provide ones effective for crystallization. Second, diabodies, particularly those stabilized by a disulfide bridge in the Fv interface, should be able to link target proteins and crystallization chaperones in a rigid and crystallizable fashion. By screening many crystallization “chaperones” and the diabodies connecting them to target proteins, one might be able to find the most suitable target-diabody-chaperone complex for generating crystals suitable for high resolution structure determination. One would not have to produce additional antibody clones against the target protein because one would only need to change one of the Fv domains that bind to the chaperone. Thousands of structures of antibody-antigen complexes have already been deposited in the Protein Data Bank and can be used as crystallization chaperones for crystallizing challenging target proteins. Crystallization chaperones are usually connected to their target proteins by flexible linkers^26^,^27^,^28^,^29^,^30^,^31^. This does not always improve the crystallization behavior of the target-chaperone fusion proteins because the great structural flexibility of the linker usually diminishes the crystallization behavior of the fusion proteins. Diabodies, particularly disulfide bridged ones, have rigid structures and can serve as ideal linkers connecting targets and crystallization chaperones.

Single particle cryo-electron microscopic analysis is becoming increasingly important in structural studies of proteins, particularly of large protein complexes. For high resolution structural analysis, averaging hundreds of thousands of images of protein particles is necessary to improve resolution. Currently, structural analysis at atomic resolution appears limited to proteins or protein complexes over ~100 kDa^32^. Diabodies can be effective tool for single particle analysis because they can link target proteins of less than ~100 kDa to larger helper proteins. The resulting “target-diabody-helper” complex should be large enough for easy and reliable averaging. Structural rigidity of the diabodies is crucial for this purpose because structural variation between images reduces the effective resolution of the structure by impairing the averaging process.

In conclusion, we have shown that diabodies have significant structural diversity. Substitution of an arginine residue and the introduction of a disulfide bridge in the Fv interface greatly reduces the structural diversity of diabodies and renders their structures more predictable. Diabodies stabilized by disulfide bridges may have broad applications in the construction of protein nano-assemblies, protein crystallization and the single particle analysis of protein cryo-EM images.

## Methods

### Preparation of diabodies

Some of the antibody genes used in this study were synthesized based on sequences deposited in databases (Supplementary Table S2). Other antibody genes were cloned in this laboratory from hybridoma cells. The antibody gene for MBP was obtained from Dr. Kossiakoff, University of Chicago. The genes for diabodies were assembled by PCR and cloned into the pAcGP67A baculovirus transfer vector (BD Biosciences). Mutations were introduced by overlap PCR. Recombinant viruses were produced according to the protocol provided by the supplier. The diabody proteins were expressed in High Five insect cells (Invitrogen) grown in SF900 medium (Invitrogen) or YSD medium^33^. Cells infected by recombinant baculoviruses were incubated at 28 °C for 3 days. Hexahistidine-tagged diabodies secreted into the medium were purified by Ni-NTA affinity chromatography (GE Healthcare). The histidine tags and inter-chain linkers of bispecific diabodies were removed by thrombin cleavage. The proteins were further purified by Superdex 200 (GE Healthcare) size exclusion chromatography using a buffer containing 20 mM Tris-HCl pH 7.5 and 200 mM NaCl. The TLR3, MBP and Repebody genes were cloned and the corresponding proteins produced as previously published^34^,^35^,^36^. In order to prepare diabody-antigen complexes, diabody proteins were mixed with the corresponding antigens. Unbound antigen was removed by Superdex 200 gel filtration chromatography.

### Crystallization and data collection

The crystallization conditions for diabodies and freezing conditions for their crystals are summarized in Supplementary Table S1. All crystals were flash-frozen in liquid nitrogen at −170 °C. Diffraction data were collected at the 7A and 5C beam lines of the Pohang Accelerator Laboratory. The package HKL2000 was used to index, integrate, and scale the diffraction data (HKL Research).

### Structure Determination and Refinement

Initial phases were calculated by the molecular replacement technique using the program PHASER^37^. Crystallographic data are summarized in Supplementary Tables S3 and S4. Atomic models were built by iterative modeling and refinement using the programs COOT and PHENIX^38^,^39^.

### Data Availability

Atomic coordinates and diffraction data have been deposited in the Protein Data Bank. The code numbers are written in Supplementary Tables S3 and S4.

## Additional Information

**How to cite this article**: Kim, J. H. *et al*. Crystal structures of mono- and bi-specific diabodies and reduction of their structural flexibility by introduction of disulfide bridges at the Fv interface. *Sci. Rep.* **6**, 34515; doi: 10.1038/srep34515 (2016).

## Supplementary Material

Supplementary Information
