# Supplementary material for: Crystal structures of mono- and bi-specific diabodies and reduction of their structural flexibility by introduction of disulfide bridges at the Fv interface
Source: Sci Rep. 2016 Sep 29;6:34515. doi: 10.1038/srep34515 (PMC5041106; doi:10.1038/srep34515)
Supplement: Supplementary Information [file srep34515-s1.pdf]

Supplementary Information

Crystal structures of mono- and bi-specific diabodies and reduction of their structural flexibility by introduction of disulfide bridges at the Fv interface.

Jin Hong Kim, Dong Hyun Song, Suk-Jun Youn, Ji Won Kim, Geunyoung Cho, Sun Chang Kim, Hayyoung Lee, Mi Sun Jin and Jie-Oh Lee

Email: misunjin@gist.ac.kr, jieoh@kaist.ac.kr

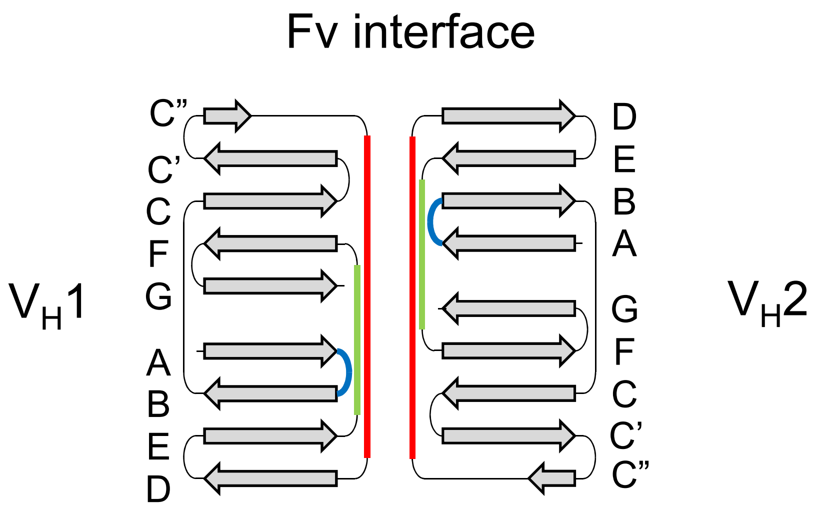

Supplementary Figure S1 | Schematic diagram of the Fv interface.
The AB, C"D and EF loops are colored in cyan, red and green, respectively.

```
V_H1
4937 - QVQLKESGPGLVRPSQSLSLTCSVTGYSITSGYYWNWIRQFPGNKLEWMGYIS YDGSNNYNPSLKGRISI
6683 - QVQLKESGPGLVRPSQSLSLTCSVTGYSITSGYYWNWIRQFPGNKLEWMGYIS YDGSNNYNPSLKGRISI
7128 - QVQLKESGPELVKPGASVKMSCKASGYTFTSYVIH WVKQRPGQGLEWIGYINPYNDGSKYNEKFKGKATL

GGGGS Linker
4937 - TRDTSKNQFFLKLNSVTTDDTATYYCARAYIGF    AYWGQGTLVTVSSGGGGSDIQMTQSPSSLSAS
6683 - TRDTSKNQFFLKLNSVTTDDTATYYCARAYIGF    AYWGQGTLVTVSSGGGGSDIVLTQPPS*VSVA
7128 - TSDKSSSTAYMDLSSLTSEDSAVYYCARYGVRGDYYAVDYWGQGTSVTVSSGGGGSDIQMTQSPSSLSAS

V_L2
4937 - VGDRVTITCRASQSVSSAVAWYQQKPGKAPKLLIYSASSLYSGVPSRFSGSRSGTDFTLTISSLQPEDFA
6683 - PGQTARISCSGDNIGSYYVHWYQQKPGQAPVLVIYEDSERPSGIPERFSGSNSGNTATLTISGTQAEDEA
7128 - VGDRVTITCRASQSVSSAVAWYQQKPGKAPKLLIYSASSLYSGVPSRFSGSRSGTDFTLTISSLQPEDFA

Interchain Linker
4937 - TYYCQQSSSSL ITFGQGTKVEIKPWLVPRGSHHHHHHSGGGGSGGGGSGGGGSGGGGLVPRGSPGEVQL
6683 - DYYCSSYDDPNFQVFGGGTKLTVKPWLVPRGSHHHHHHSGGGGSGGGGSGGGGSGGGGLVPRGSPGQVQL
7128 - TYYCQQSSSSL ITFGQGTKVEIKPWLVPRGSHHHHHHSGGGGSGGGGSGGGGSGGGGLVPRGSPGEVQL

V_H2
4937 - VESGGGLVQPGGSLRLSCAASGFNFSSSSIH  WVRQAPGKGLEWVASISS SSGSTSYADSVKGRFTIS
6683 - QQSGPGLVKPSQTLSLTCAISGDSVSSNSAAWGWIRQSPGRGLEWLGIIQKRSKWYNNYAVKVKSKITIN
7128 - VESGGGLVQPGGSLRLSCAASGFNFSSSSIH  WVRQAPGKGLEWVASISS SSGSTSYADSVKGRFTIS

GGGGS Linker
4937 - ADTSKNTAYLQMNSLTAEDTAVYYCARGYYYTGLWYPYAMYEFGMDYWGQGTLVTVSSGGGGSDIQLTQS
6683 - PDTSKNQFSLQLSSVTPEDTAVYYCARYSYPFYSI          DYWGQGTLVTVSSGGGGSDIQLTQS
7128 - ADTSKNTAYLQMNSLTAEDTAVYYCARYGHWSWGRWWNYWVAL  DYWGQGTLVTVSSGGGGSDIVMTQS

V_L1
4937 - TSSLPASLGDRVTISCRAGQDISNHLNWYQQKPDGTVKLLIYYTSRLHSGVPSRFSGSGSGTDYSLTISN
6683 - TSSLPASLGDRVTISCRAGQDISNHLNWYQQKPDGTVKLLIYYTSRLHSGVPSRFSGSGSGTDYSLTISN
7128 - QKFMSTSAGDRVSITCKASQNVRTAVAWYQQKPGQSPKALIYLASNRHTGVPDRFTGSGSGTDFTLTISN

4937 - LEQEDIATYFCQQGNTLPWTFGGGSKLEIK
6683 - LEQEDIATYFCQQGNTLPWTFGGGSKLEIK
7128 - VQSEDLADYFCLQHWSYPYTFGGGTKLEIK
```

Supplementary Figure S2 | Sequence alignment of the bispecific diabodies 4937, 6683 and 7128. The amino acids are numbered according to the Kabat numbering scheme. Amino acids that share the same residue number are marked with a bar above the sequence.

A

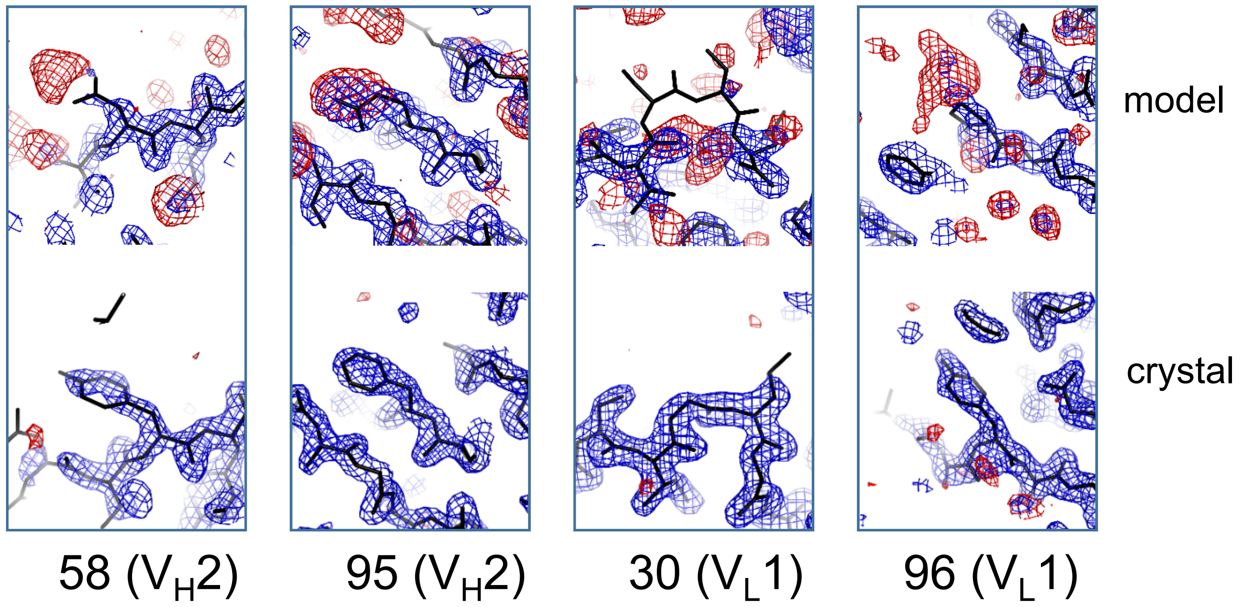

B

```
                 10        20        30        40        50        60
                  •         •         •         •         •         •
VH1 - EVQLVESGGG LVQPGGSLRL SCAASGFTFR NSAMHWVRQA PGKGLEWVSS IWYSGSNTYYA
VH2 - EVQLVQSGAE VKKPGESLKI SCKGSGYSFT NYWVGWVRQM PGKGLEWMGF IDPSDSYTNYA

                 70        80          90          100        110
                  •         •           •          •           •
VH1 - DSVKGRFTIS RDNSKNTLYL QMNSLRAEDTAVY YCARFAGGWGAY  DVWGQGTLVT VSS
VH2 - PKFQGKVTIS ADKSISTAYL QMSSLTASDTAMY YCARELYQGYMDTF DSWGQGTLVT VSS

                  10        20          30        40        50        60
                   •         •           •         •         •         •
VL1  - DIVLTQSPAT LSLSPGERAT LSCRASQSVSS NYLAWYQQKP GQAPRLLIYD SSSRATGVPA
VL2  - DIQMTQSPSS LSASVGDRVT ITCRASQ SIG LYLAWYQQKP GKAPKLLIYA ASSLQSGVPS

                  70        80        90       100
                   •         •         •         •
VL1  - RFSGSGSGTD FTLTISSLEP EDFAVYYCHQ YSDISPTFGQ GTKVEIK
VL2  - RFSGSGSGTD FTLTISSLQP EDFATYYCQQ GNTLSYTFGQ GTKVEIK
```

Supplementary Figure S3 | Chain shuffling of the bispecific diabody, 6277. (**A**) The intended structures that does not fit to the electron density map are shown in the upper panels. The correct and shuffled crystal structures are shown in the lower panels. The 2Fo-Fc and the Fo-Fc maps contoured at 1.0 and -2.0 σ, respectively, are drawn in blue and red, respectively. The residue numbers are written at the bottom. (**B**) The sequences of the V_H1 and V_H2 regions of the 6277 diabody are aligned. The amino acids are numbered according to the Kabat numbering scheme. The residues whose structures are shown in (**A**) are colored in red. Amino acids that share the same residue number are marked with a bar above the sequence.

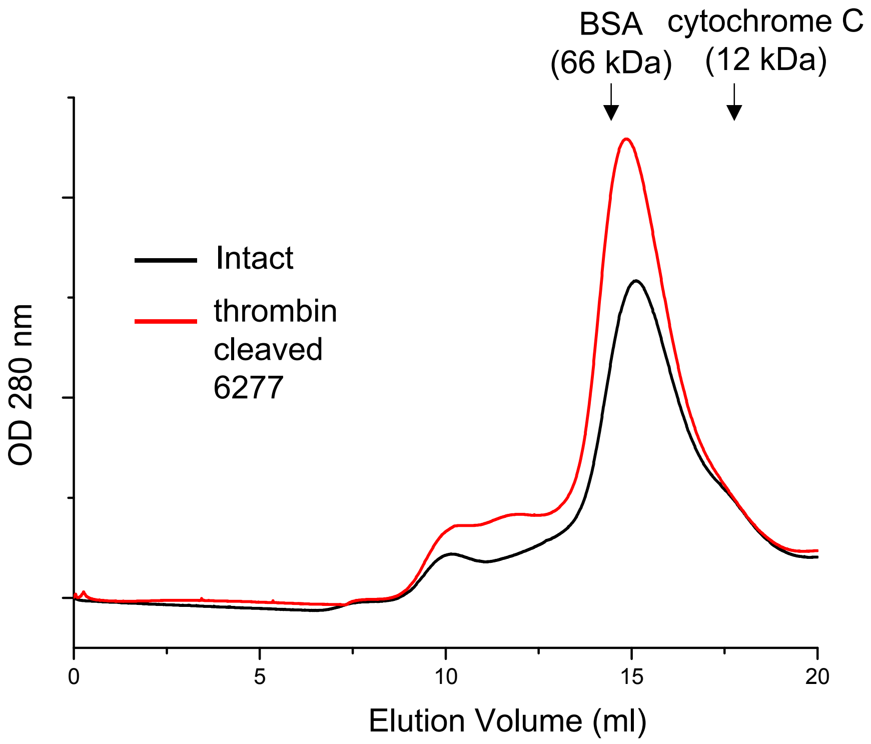

Supplementary Figure S4 | Size exclusion chromatography of the 6277 diabody.
The intact and thrombin-cleaved forms of the 6277 diabody were injected into a Superdex 200 gel filtration chromatography column. Elution volumes of Bovine Serum Albumin (BSA) and cytochrome C proteins are marked. Molecular weights of the intact and thrombin cleaved forms of 6277 are 55 and 51 kDa, respectively.

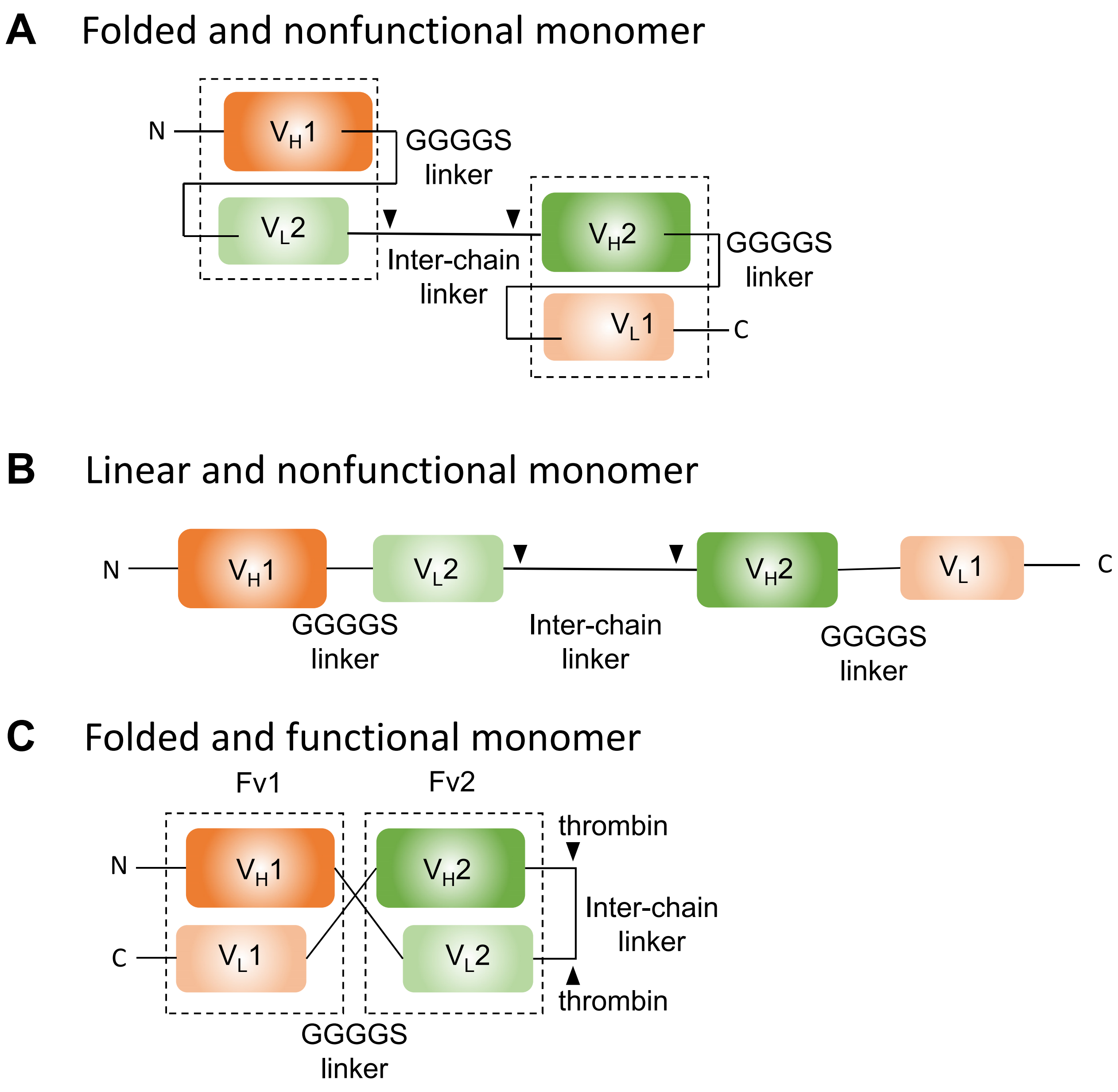

Supplementary Figure S5 | Schematic models of the intact 6266 diabody.
(A) Folded and nonfunctional monomer. This model is impossible because the "GGGGS" linker is too short to connect the V_H C-terminus and the V_L N-terminus (B) Linear and nonfunctional monomer. This is another unlikely model because the V_H and V_L domains do not dissociate in physiological conditions. (C) Folded and functional monomer. This is our favored model. Thrombin cleavage may initiate chain shuffling for some diabodies.

```
                                            VH
             10    AB        20        30        40        50          60   C"D
6052 - EVQLVESGGGLVQPGGSLRLSCAASGFTFRNSAMHWVRQAPGKGLEWVSSIWYSGSNTYYADSVKGRFTI
4921 -              -                                               -  -
4922 -              -                                               -  -
4960 -              -                                               -  -
4970 -              -                                               C  -
4971 -              C                                               -  C
4967 - QVQLVQSGAEVVKPGASVKLSCKASGYIFTSYYMYWVKQAPGQGLEWIGEINPSNGDTNFNEKFKSKATL
4342 - EVQLVESGGGLVQPGGSLRLSCAASGYTFTNYGMNWVRQAPGKGLEWVGWINTYTGEPTYAADFKRRFTF

                          VH                                  GGGGS     VL
                                                              linker
       70        80     EF   90       100             110               10
6052 - SRDNSKNTLYLQMNSLTAEDTAVYYCARFAGGWGAY    DVWGQGTLVTVSSGGGGSDIVLTQSPATLS
4921 -                 F -                                  -
4922 -                 I -                                  -
4960 -                 - C                                  -
4970 -                 - -                                  C
4971 -                 - -                                  -
4967 - TVDKSASTAYMELSSLTSCDTAVYYCTRSDGRNDM     DSWGQGTLVTVSSGGGGSDIVLTQSPATLS
4932 - SLDTSKSTAYLQMNSLTACDTAVYYCAKYPHYYGSSHWYFDVWGQGTLVTVSSGGGGSDIQMTQSPSSLS

                                            VL
              20            30        40        50         60        70
6052 - LSPGERATLSCRASQS   VSSNYLAWYQQKPGQAPRLLIYDSSSRATGVPARFSGSGSGTDFTLTISSL
4921 -
4922 -
4960 -
4970 -
4971 -
4967 - VSPGERATISCRASQRVSSSTYSYMHWYQQKPGQPPKLLIKYASNLESGVPARFSGSGSGTDFTLTISSV
4342 - ASVGDRVTITCSASQ    DISNYLNWYQQKPGKAPKVLIYFTSSLHSGVPSRFSGSGSGTDFTLTISSL

                    VL
        80         90         100
6052 - EPEDFAVYYCHQYSDISPTFGQGTKVEIK
4921 -
4922 -
4960 -
4970 -
4971 -
4967 - EPEDFATYYCQHSWEIPPTFGGGTKLEIK
4342 - QPEDFATYYCQQYSTVPWTFGQGTKVEIK
```

Supplementary Figure S6 | Sequence alignment of the monospecific diabodies. The amino acids are numbered according to the Kabat numbering scheme. Amino acids that share the same residue number are marked with a bar above the sequence.

```
                                        VH1
              10    AB     20        30        40         50        60   C"D   70
6851 – EVQLVESGGGLVQPGGSLRLSCAASGFTFRNSAMHWVRQAPGKGLEWVSSIWYSGSNTYYADSVKGRFTIS
6857 – EVQLVESGGGLVQPGGSLRLSCAASGFTFRNSAMHWVRQAPGKGLEWVSSIWYSGSNTYYADSVKGRFTIS
6886 – EVQLVESGGGLVQPGGSLRLSCAASGFTFRNSAMHWVRQAPGKGLEWVSSIWYSGSNTYYADSVKGRFTIS

                                                      GGGGS
                                                      Linker
              80    EF         90      100          110            10
6851 – RDNSKNTLYLQMNSLTACDTAVYYCARFAGGWGAYDVWGQGTLVTVSSGGGGSDIQMTQSPSSLSASVGDR
6857 – RDNSKNTLYLQMNCLTAEDTAVYYCARFAGGWGAYDVWGQGTLVTVSSGGGGSDIQMTQSPSSLSASVGDR
6886 – RDNSKNTLYLQMNSLCAEDTAVYYCARFAGGWGAYDVWGQGTLVTVSSGGGGSDIQMTQSPSSLSASVGDR

                                        VL2
        20         30        40        50        60        70        80
6851 – VTITCRASQSVSSAVAWYQQKPGKAPKLLIYSASSLYSGVPSRFSGSRSGTDFTLTISSLQPEDFATYYCQ
6857 – VTITCRASQSIGLYLAWYQQKPGKAPKLLIYAASSLQSGVPSRFSGSGSGTDFTLTISSLQPEDFATYYCQ
6886 – VTITCRASQSIGLYLAWYQQKPGKAPKLLIYAASSLQSGVPSRFSGSGSGTDFTLTISSLQPEDFATYYCQ

                                  Interchain Linker
       90        100                                                       10
6851 – QSSYIPVTFGQGTKVEIKPWLVPRGSHHHHHHSGGGGSGGGGSGGGGSGGGGLVPRGSPGEVQLVESGGGL
6857 – QGNTLSYTFGQGTKVEIKPWLVPRGSHHHHHHSGGGGSGGGGSGGGGSGGGGLVPRGSPGEVQLVQSGAEV
6886 – QGNTLSYTFGQGTKVEIKPWLVPRGSHHHHHHSGGGGSGGGGSGGGGSGGGGLVPRGSPGEVQLVQSGAEV

                                        VH2
         AB      20        30        40         50         60   C"D    70        80
6851 – VQPGGSLRLSCAASGFNFSSSYIHWVRQAPGKGLEWVAYISSYSGYTSYADSVKGRFTISADTSKNTAYLQ
6857 – KKPGESLKISCKGSGYSFTNYWVGWVRQMPGKGLEWMGFIDPSDSYTNYADSVKGRFTISADKSISTAYLQ
6886 – KKPGESLKISCKGSGYSFTNYWVGWVRQMPGKGLEWMGFIDPSDSYTNYADSVKGRFTISADKSISTAYLQ

                                              GGGGS
                                              Linker
          EF       90       100           110                 10         20
6851 – MNSLTACDTAVYYCARTPWWYWSGL DYWGQGTLVTVSSGGGGSDIVLTQSPATLSLSPGERATLSCRASQ
6857 – MNCLTASDTAMYYCARELYQGYMDTFDSWGQGTLVTVSSGGGGSDIVLTQSPATLSLSPGERATLSCRASQ
6886 – MNSLCASDTAMYYCARELYQGYMDTFDSWGQGTLVTVSSGGGGSDIVLTQSPATLSLSPGERATLSCRASQ

                                        VL1
         30        40        50        60        70         80         90
6851 – SVSSNYLAWYQQKPGQAPRLLIYDSSSRATGVPARFSGSGSGTDFTLTISSLEPEDFAVYYCHQYSDISPT
6857 – SVSSNYLAWYQQKPGQAPRLLIYDSSSRATGVPARFSGSGSGTDFTLTISSLEPEDFAVYYCHQYSDISPT
6886 – SVSSNYLAWYQQKPGQAPRLLIYDSSSRATGVPARFSGSGSGTDFTLTISSLEPEDFAVYYCHQYSDISPT

        100
6851 – FGQGTKVEIK
6857 – FGQGTKVEIK
6886 – FGQGTKVEIK
```

Supplementary Figure S7 | Sequence alignment of the bispecific diabodies with disulfide bridges, 6851, 6857 and 6886. The amino acids are numbered according to the Kabat numbering scheme. Amino acids that share the same residue number are marked with a bar above the sequence.

```
                                   VH1
6887 - EVQLVESGGGLVQPGGSLRLSCAASGFTFRNSAMHWVRQAPGKGLEWVSSIWYSGSNTYYADSVKGRFTIS
6888 - EVQLVESGGGLVQPGGSLRLSCAASGFTFRNSAMHWVRQAPGKGLEWVSSIWYSGSNTYYADSVKGRFTIS

                                                  GGGGS Linker
6887 - RDNSKNTLYLQMNSLTACDTAVYYCARFAGGWGAYDVWGQGTLVTVSSGGGGSDIQMTQSPSSLSASVGDR
6888 - RDNSKNTLYLQMNCLTAEDTAVYYCARFAGGWGAYDVWGQGTLVTVSSGGGGSDIQMTQSPSSLSASVGDR

                                   VL2
6887 - VTITCRASQSIGLYLAWYQQKPGKAPKLLIYAASSLQSGVPSRFSGSGSGTDFTLTISSLQPEDFATYYCQ
6888 - VTITCRASQSIGLYLAWYQQKPGKAPKLLIYAASSLQSGVPSRFSGSGSGTDFTLTISSLQPEDFATYYCQ

                              Interchain Linker
6887 - QGNTLSYTFGQGTKVEIKPWLVPRGSHHHHHHSGGGGSGGGGSGGGGSGGGGLVPRGSPGEVQLVQSGAEV
6888 - QGNTLSYTFGQGTKVEIKPWLVPRGSHHHHHHSGGGGSGGGGSGGGGSGGGGLVPRGSPGEVQLVQSGAEV

                                   VH2
6887 - KKPGESLKISCKGSGYSFTNYWVGWVRQMPGKGLEWMGFIDPSDSYTNYADSVKGRFTISADKSISTAYLQ
6888 - KKPGESLKISCKGSGYSFTNYWVGWVRQMPGKGLEWMGFIDPSDSYTNYADSVKGRFTISADKSISTAYLQ

                                        GGGGS Linker
6887 - MNSLTACDTAMYYCARELYQGYMDTFDSWGQGTLVTVSSGGGGSDIVLTQSPATLSLSPGERATLSCRASQ
6888 - MNSLCASDTAMYYCARELYQGYMDTFDSWGQGTLVTVSSGGGGSDIVLTQSPATLSLSPGERATLSCRASQ

                                   VL1
6887 - SVSSNYLAWYQQKPGQAPRLLIYDSSSRATGVPARFSGSGSGTDFTLTISSLEPEDFAVYYCHQYSDISPT
6888 - SVSSNYLAWYQQKPGQAPRLLIYDSSSRATGVPARFSGSGSGTDFTLTISSLEPEDFAVYYCHQYSDISPT

6887 - FGQGTKVEIK
6888 - FGQGTKVEIK
```

Supplementary Figure S8 | Sequence alignment of the bispecific diabodies with disulfide bridges, 6887 and 6888. The amino acids are numbered according to the Kabat numbering scheme. Amino acids that share the same residue number are marked with a bar above the sequence.

```
V_H1
          10  AB     20        30        40        50           60  C"D     70
6889 - EVQLVESGGGLVQPGGSLRLSCAASGFTFRNSAMHWVRQAPGKGLEWVSSIWYSGSNTYYADSVKGRFTIS
6890 - EVQLVESGGGLVQPGGSLRLSCAASGFTFRNSAMHWVRQAPGKGLEWVSSIWYSGSNTYYADSVKGRFTIS
6891 - EVQLVESGGGLVQPGGSLRLSCAASGFTFRNSAMHWVRQAPGKGLEWVSSIWYSGSNTYYADSVKGRFTIS

                                               GGGGS
                                               Linker
          80     EF      90       100       110           10
6889 - RDNSKNTLYLQMNSLCAEDTAVYYCARFAGGWGAYDVWGQGTLVTVSSGGGGSDIQMTQSPSSLSASVGDR
6890 - RDNSKNTLYLQMNSLCAEDTAVYYCARFAGGWGAYDVWGQGTLVTVSSGGGGSDIQMTQSPSSLSASVGDR
6891 - RDNSKNTLYLQMNSLTACDTAVYYCARFAGGWGAYDVWGQGTLVTVSSGGGGSDIQMTQSPSSLSASVGDR

V_L2
   20        30        40        50        60        70        80
6889 - VTITCRASQSIGLYLAWYQQKPGKAPKLLIYAASSLQSGVPSRFSGSGSGTDFTLTISSLQPEDFATYYCQ
6890 - VTITCRASQSIGLYLAWYQQKPGKAPKLLIYAASSLQSGVPSRFSGSGSGTDFTLTISSLQPEDFATYYCQ
6891 - VTITCRASQSIGLYLAWYQQKPGKAPKLLIYAASSLQSGVPSRFSGSGSGTDFTLTISSLQPEDFATYYCQ

Interchain Linker
  90       100                                                        10
6889 - QGNTLSYTFGQGTKVEIKPWLVPRGSHHHHHHSGGGGSGGGGSGGGGSGGGGLVPRGSPGEVQLVQSGAEV
6890 - QGNTLSYTFGQGTKVEIKPWLVPRGSHHHHHHSGGGGSGGGGSGGGGSGGGGLVPRGSPGEVQLVQSGAEV
6891 - QGNTLSYTFGQGTKVEIKPWLVPRGSHHHHHHSGGGGSGGGGSGGGGSGGGGLVPRGSPGEVQLVQSGAEV

V_H2
  AB     20        30        40        50          60  C"D     70        80
6889 - KKPGESLKISCKGSGYSFTNYWVGWVRQMPGKGLEWMGFIDPSDSYTNYADSVKGRFTISADKSISTAYLQ
6890 - KKPGESLKISCKGSGYSFTNYWVGWVRQMPGKGLEWMGFIDPSDSYTNYADSVKGRFTISADKSISTAYLQ
6891 - KKPGESLKISCKGSGYSFTNYWVGWVRQMPGKGLEWMGFIDPSDSYTNYADSVKGRFTISADKSISTAYLQ

                                      GGGGS
                                      Linker
    EF      90        100          110                10        20
6889 - MNCLTASDTAMYYCARELYQGYMDTFDSWGQGTLVTVSSGGGGSDIVLTQSPATLSLSPGERATLSCRASQ
6890 - MNSLTACDTAMYYCARELYQGYMDTFDSWGQGTLVTVSSGGGGSDIVLTQSPATLSLSPGERATLSCRASQ
6891 - MNSLCASDTAMYYCARELYQGYMDTFDSWGQGTLVTVSSGGGGSDIVLTQSPATLSLSPGERATLSCRASQ

V_L1
   30        40        50        60        70        80        90
6889 - SVSSNYLAWYQQKPGQAPRLLIYDSSSRATGVPARFSGSGSGTDFTLTISSLEPEDFAVYYCHQYSDISPT
6890 - SVSSNYLAWYQQKPGQAPRLLIYDSSSRATGVPARFSGSGSGTDFTLTISSLEPEDFAVYYCHQYSDISPT
6891 - SVSSNYLAWYQQKPGQAPRLLIYDSSSRATGVPARFSGSGSGTDFTLTISSLEPEDFAVYYCHQYSDISPT

   100
6889 - FGQGTKVEIK
6890 - FGQGTKVEIK
6891 - FGQGTKVEIK
```

Supplementary Figure S9 | Sequence alignment of the bispecific diabodies with disulfide bridges, 6889-91. The amino acids are numbered according to the Kabat numbering scheme. Amino acids that share the same residue number are marked with a bar above the sequence.

```
V_H1
6892 - EVQLVESGGGLVQPGGSLRLSCAASGFTFRNSAMHWVRQAPGKGLEWVSSIWYSGSNTYYADSVKGRFTIS
6893 - EVQLVESGGGLVQPGGSLRLSCAASGFTFRNSAMHWVRQAPGKGLEWVSSIWYSGSNTYYADSVKGRFTIS

GGGGS Linker
6892 - RDNSKNTLYLQMNSLTCEDTAVYYCARFAGGWGAYDVWGQGTLVTVSSGGGGSDIQMTQSPSSLSASVGDR
6893 - RDNSKNTLYLQMNSLTACDTAVYYCARFAGGWGAYDVWGQGTLVTVSSGGGGSDIQMTQSPSSLSASVGDR

V_L2
6892 - VTITCRASQSIGLYLAWYQQKPGKAPKLLIYAASSLQSGVPSRFSGSGSGTDFTLTISSLQPEDFATYYCQ
6893 - VTITCRASQSIGLYLAWYQQKPGKAPKLLIYAASSLQSGVPSRFSGSGSGTDFTLTISSLQPEDFATYYCQ

Interchain Linker
6892 - QGNTLSYTFGQGTKVEIKPWLVPRGSHHHHHHSGGGGSGGGGSGGGGSGGGGLVPRGSPGEVQLVQSGAEV
6893 - QGNTLSYTFGQGTKVEIKPWLVPRGSHHHHHHSGGGGSGGGGSGGGGSGGGGLVPRGSPGEVQLVQSGAEV

V_H2
6892 - KKPGESLKISCKGSGYSFTNYWVGWVRQMPGKGLEWMGFIDPSDSYTNYADSVKGRFTISADKSISTAYLQ
6893 - KKPGESLKISCKGSGYSFTNYWVGWVRQMPGKGLEWMGFIDPSDSYTNYADSVKGRFTISADKSISTAYLQ

GGGGS Linker
6892 - MNSLTACDTAMYYCARELYQGYMDTFDSWGQGTLVTVSSGGGGSDIVLTQSPATLSLSPGERATLSCRASQ
6893 - MNSLTCSDTAMYYCARELYQGYMDTFDSWGQGTLVTVSSGGGGSDIVLTQSPATLSLSPGERATLSCRASQ

V_L1
6892 - SVSSNYLAWYQQKPGQAPRLLIYDSSSRATGVPARFSGSGSGTDFTLTISSLEPEDFAVYYCHQYSDISPT
6893 - SVSSNYLAWYQQKPGQAPRLLIYDSSSRATGVPARFSGSGSGTDFTLTISSLEPEDFAVYYCHQYSDISPT

6892 - FGQGTKVEIK
6893 - FGQGTKVEIK
```

Supplementary Figure S10 | Sequence alignment of the bispecific diabodies with disulfide bridges, 6892 and 6893. The amino acids are numbered according to the Kabat numbering scheme. Amino acids that share the same residue number are marked with a bar above the sequence.

```
                                       VH1
           10   AB    20        30        40        50        60   C"D
6861 - EVQLVESGGGLVQCGGSLRLSCAASGFTFRNSAMHWVRQAPGKGLEWVSSIWYSGSNTYYADSVKGRFTI
6875 - EVQLVESGGGLVQPGGSLRLSCAASGFTFRNSAMHWVRQAPGKGLEWVSSIWYSGSNTYYADSVKGRFTI
4968 - EVQLVESGGGLVQPGGSLRLSCAASGYTFTNYGMNWVRQAPGKGLEWVGWINTYTGEPTYAADFKRRFTF

                                                   GGGGS
                                                   Linker
70        80   EF      90       100       110              10
6861 - SRDNSKNTLYLQMNSLTAEDTAVYYCARFAGGWGAY     DVWGQGTLVTVSSGGGGSDIQMTQSPASLSA
6875 - SRDNSKNTLYLQMNCLTAEDTAVYYCARFAGGWGAY     DVWGQGTLVTVSSGGGGSDIQMTQSPASLSA
4968 - SLDTSKSTAYLQMNSLTACDTAVYYCAKYPHYYGSSHWYFDVWGQGTLVTVSSGGGGSDIVLTQSPATLSV

                                       VL2
         20         30       40        50        60        70        80
6861 - SVGETVTITCRASG     NIHNYLAWYQQKQGKSPQLLVYNAKTLADGVPSRFSGSESGTQYSLKINSLQP
6875 - SVGETVTITCRASG     NIHNYLAWYQQKQGKSPQLLVYNAKTLADGVPSRFSGSESGTQYSLKINSLQP
4968 - SPGERATISCRASQRVSSSTYSYMHWYQQKPGQPPKLLIKYASNLESGVPARFSGSGSGTDFTLTISSVEP

                                   Interchain Linker
          90        100
6861 - EDFGSYYCQHFWSTPFTFGSGTKLELKPWLVPRGSHHHHHHSGGGGSGGGGSGGGGSGGGGLVPRGSPG*V
6875 - EDFGSYYCQHFWSTPFTFGSGTKLELKPWLVPRGSHHHHHHSGGGGSGGGGSGGGGSGGGGLVPRGSPG*V
4968 - EDFATYYCQHSWEIPPTFGGGTKLEIKPWLVPRGSHHHHHHSGGGGSGGGGSGGGGSGGGGLVPRGSPGQV

                                       VH2
          10  AB    20        30        40        50        60  C"D    70
6861 - QLQQPGAELVQPGTSVRLSCKASGYIFTTYWIHWVKQRPGQGLEWIGEINPNNGRINYADSVCGRFTLTVD
6875 - QLQQPGAELVQPGTSVRLSCKASGYIFTTYWIHWVKQRPGQGLEWIGEINPNNGRINYADSVKGRFTLTVD
4968 - QLVQSGAEVVKPGASVKLSCKASGYIFTSYYMYWVKQAPGQGLEWIGEINPSNGDTNFNEKFKSKATLTVD

                                             GGGGS
                                             Linker
          80    EF      90        100       110              10        20
6861 - KSSSTAYMQMNSLTAEDSAVYYCTRVGVMITTFPYWGQGTLVTVSSGGGGSDIVLTQSPATLSLSPGERAT
6875 - KSSSTAYMQMNCLTAEDSAVYYCTRVGVMITTFPYWGQGTLVTVSSGGGGSDIVLTQSPATLSLSPGERAT
4968 - KSASTAYMELSSLTSCDTAVYYCTRSDGRNDM DSWGQGTLVTVSSGGGGSDIQMTQSPSSLSASVGDRVT

                                       VL1
           30        40        50        60        70        80        90
6861 - LSCRASQSVSSSNYLAWYQQKPGQAPRLLIYDSSSRATGVPARFSGSGSGTDFTLTISSLEPEDFAVYYCHQ
6875 - LSCRASQSVSSSNYLAWYQQKPGQAPRLLIYDSSSRATGVPARFSGSGSGTDFTLTISSLEPEDFAVYYCHQ
4968 - ITCSASQ DISNYLNWYQQKPGKAPKVLIYFTSSLHSGVPSRFSGSGSGTDFTLTISSLQPEDFATYYCQQ

           100
6861 - YSDISPTFGQGTKVEIK
6875 - YSDISPTFGQGTKVEIK
4968 - YSTVPWTFGQGTKVEIK
```

Supplementary Figure S11 | Sequence alignment of the bispecific diabodies with disulfide bridges, 6861, 6875 and 4968. The amino acids are numbered according to the Kabat numbering scheme. Amino acids that share the same residue number are marked with a bar above the sequence.

```
V_H1
            10  AB     20        30          40        50          60  C"D
4977 - QVQLKESGPGLVRCSQSLSLTCSVTGYSITSGYYWNWIRQFPGNKLEWMGYISYDGSNNYNPSLKGRISI
4979 - QVQLKESGPGLVRPSQSLSLTCSVTGYSITSGYYWNWIRQFPGNKLEWMGYISYDGSNNYNPSLCGRISI

                                                GGGGS
                                                Linker
70        80   EF      90       100      110                     10
4977 - TRDTSKNQFFLKLNSVTTDDTATYYCARAYIGF*AYWGQGTLVTVSSGGGGSDIVLTQPPS*VSVAPGQTA
4979 - TRDTSKNQFFLKLNSVTTDDTATYYCARAYIGF*AYWGQGTLVTVSSGGGGSDIVLTQPPS*VSVAPGQTA

V_L2
20        30        40        50        60        70        80        90
4977 - RISCSGDNIGSYYVHWYQQKPGQAPVLVIYEDSERPSGIPERFSGSNSGNTATLTISGTQAEDEADYYCSS
4979 - RISCSGDNIGSYYVHWYQQKPGQAPVLVIYEDSERPSGIPERFSGSNSGNTATLTISGTQAEDEADYYCSS

                  Interchain Linker
          100                                                                 10
4977 - YDDPNFQVFGGGTKLTVKPWLVPRGSHHHHHHSGGGGSGGGGSGGGGSGGGGLVPRGSPGQVQLQQSGPGL
4979 - YDDPNFQVFGGGTKLTVKPWLVPRGSHHHHHHSGGGGSGGGGSGGGGSGGGGLVPRGSPGQVQLQQSGPGL

V_H2
 AB      20        30          40        50          60  C"D      70
4977 - VKPSQTLSLTCAISGDSVSSNSAAWGWIRQSPGRGLEWLGIIQKRSKWYNNYAVKVCSKITINPDTSKNQF
4979 - VKCSQTLSLTCAISGDSVSSNSAAWGWIRQSPGRGLEWLGIIQKRSKWYNNYAVKVKSKITINPDTSKNQF

                                          GGGGS
                                          Linker
 80    EF     90       100       110                  10        20
4977 - SLQLSSVTPEDTAVYYCARYSYPFYSIDYWGQGTLVTVSSGGGGSDIQLTQSTSSLPASLGDRVTISCRAG
4979 - SLQLSSVTPEDTAVYYCARYSYPFYSIDYWGQGTLVTVSSGGGGSDIQLTQSTSSLPASLGDRVTISCRAG

V_L1
   30        40        50        60        70        80        90
4977 - QDISNHLNWYQQKPDGTVKLLIYYTSRLHSGVPSRFSGSGSGTDYSLTISNLEQEDIATYFCQQGNTLPWT
4979 - QDISNHLNWYQQKPDGTVKLLIYYTSRLHSGVPSRFSGSGSGTDYSLTISNLEQEDIATYFCQQGNTLPWT

   100
4977 - FGGGSKLEIK
4979 - FGGGSKLEIK
```

Supplementary Figure S12 | Sequence alignment of the bispecific diabodies with disulfide bridges, 4977 and 4979. The amino acids are numbered according to the Kabat numbering scheme. Amino acids that share the same residue number are marked with a bar above the sequence.

```
                                         VH1
          10   AB   20        30           40         50          60  C"D
7117 - QVQLKESGPGLVRPSQSLSLTCSVTGYSITSGYYWNWIRQFPGNKLEWMGYIS YDGSNNYNPSLCNRISI
7609 - EVQLQQSGSELVKPGASVKMSCKASGYTFTNYVTH WVKEKPGQGLEWIGYINPYNEGTKYNEKFKGKATL
7613 - EVQLQQSGSELVKPGASVKMSCKASGYTFTNYVTH WVKEKPGQGLEWIGYINPYNEGTKYNEKFKGKATL

                                                   GGGGS
                                                   Linker
70        80     EF       90       100      110                 10
7117 - TRDTSKNQFFLKLNSVTTDDTATYYCARAYIGF*AYWGQGTLVTVSAGGGGSDIQMTQSPSSLSASVGDRV
7609 - TSDKSSSTAYMDLSCLTSEDSAVYYCARGGLKAMDYWGQGTSVTVSSGGGGSDIQMTQSPSSLSASVGDRV
7613 - TSDKSSSTAYMDLSSLTSCDSAVYYCARGGLKAMDYWGQGTSVTVSSGGGGSDIQMTQSPSSLSASVGDRV

                                         VL2
20        30        40        50         60         70        80          90
7117 - TITCRASQSVSSAVAWYQQKPGKAPKLLIYSASSLYSGVPSRFSGSRSGTDFTLTISSLQPEDFATYYCQQ
7609 - TITCRASQASYSSVAWYQQKPGKAPKLLIYAASYLYSGVPSRFSGSGSGTDFTLTISSLQPEDFATYYCQS
7613 - TITCRASQASYSSVAWYQQKPGKAPKLLIYAASYLYSGVPSRFSGSGSGTDFTLTISSLQPEDFATYYCQS

                                   Interchain Linker
         100                                                                10
7117 - SSSSLITFGQGTKVEIKPWLVPRGSHHHHHHSGGGGSGGGGSGGGGSGGGGLVPRGSPGEVQLVESGGGLV
7609 - SASP*ATFGQGTKVEIKPWLVPRGSHHHHHHSGGGGSGGGGSGGGGSGGGGLVPRGSPGEVQLVESGGGLV
7613 - SASP*ATFGQGTKVEIKPWLVPRGSHHHHHHSGGGGSGGGGSGGGGSGGGGLVPRGSPGEVQLVESGGGLV

                                         VH2
 AB     20        30         40         50          60  C"D    70         80
7117 - QCGGSLRLSCAASGFNFSSSSIHWVRQAPGKGLEWVASISSSSGSTSYADSVKGRFTISADTSKNTAYLQM
7609 - QPGGSLRLSCAASGFDIYDDDIHWVRQAPGKGLEWVAYIAPSYGYTDYADSVKGRFTISADTSKNTAYLQM
7613 - QPGGSLRLSCAASGFDIYDDDIHWVRQAPGKGLEWVAYIAPSYGYTDYADSVKGRFTISADTSKNTAYLQM

                                                GGGGS
                                                Linker
  EF       90        100                 110                     10        20
7117 - NSLRAEDTAVYYCARYGHWSWGRWWNYWVALDYWGQGTLVTVSSGGGGSDIQLTQSTSSLPASLGDRVTIS
7609 - NCLTAEDTAVYYCSRSSDASYSYSAM     DYWGQGTLVTVSSGGGGSDIQLTQSPASLAVSLGQRATIS
7613 - NSLTACDTAVYYCSRSSDASYSYSAM     DYWGQGTLVTVSSGGGGSDIQLTQSPASLAVSLGQRATIS

                                         VL1
            30         40         50         60         70          80
7117 - CRAGQ    DISNHLNWYQQKPDGTVKLLIYYTSRLHSGVPSRFSGSGSGTDYSLTISNLEQEDIATYFCQ
7609 - CRASKSVSTSGYSYMHWYQQKPGQPPKLLIYLASNLESGVPARFSGSGSGTDFTLNIHPVEEEDAATYYCQ
7613 - CRASKSVSTSGYSYMHWYQQKPGQPPKLLIYLASNLESGVPARFSGSGSGTDFTLNIHPVEEEDAATYYCQ

90         100
7117 - QGNTLPWTFGGGSKLEIK
7609 - HSREFPWTFGGGTKLEIK
7613 - HSREFPWTFGGGTKLEIK
```

Supplementary Figure S13 | Sequence alignment of the bispecific diabodies with disulfide bridges, 7117, 7609 and 7613. The amino acids are numbered according to the Kabat numbering scheme. Amino acids that share the same residue number are marked with a bar above the sequence.

```
                     VH1
            10   AB   20         30         40         50         60  C"D
7614 - EVQLQQSGSELVKCGASVKMSCKASGYTFTNYVTHWVKEKPGQGLEWIGYINPYNEGTKYNEKFKGKATL
7615 - EVQLQQSGSELVKPGASVKMSCKASGYTFTNYVTHWVKEKPGQGLEWIGYINPYNEGTKYNEKFCGKATL

                                                   GGGGS Linker
70          80   EF      90        100       110               10
7614 - TSDKSSSTAYMDLSSLTSEDSAVYYCARGGLKAMDYWGQGTSVTVSSGGGGSDIQMTQSPSSLSASVGDRV
7615 - TSDKSSSTAYMDLSSLTSEDSAVYYCARGGLKAMDYWGQGTSVTVSSGGGGSDIQMTQSPSSLSASVGDRV

                     VL2
20          30         40         50         60         70         80        90
7614 - TITCRASQASYSSVAWYQQKPGKAPKLLIYAASYLYSGVPSRFSGSGSGTDFTLTISSLQPEDFATYYCQS
7615 - TITCRASQASYSSVAWYQQKPGKAPKLLIYAASYLYSGVPSRFSGSGSGTDFTLTISSLQPEDFATYYCQS

                     Interchain Linker
          100                                                            10
7614 - SASP*ATFGQGTKVEIKPWLVPRGSHHHHHHSGGGGSGGGGSGGGGSGGGGLVPRGSPGEVQLVESGGGLV
7615 - SASP*ATFGQGTKVEIKPWLVPRGSHHHHHHSGGGGSGGGGSGGGGSGGGGLVPRGSPGEVQLVESGGGLV

                     VH2
AB        20         30         40         50         60  C"D   70         80
7614 - QPGGSLRLSCAASGFDIYDDDIHWVRQAPGKGLEWVAYIAPSYGYTDYADSVCGRFTISADTSKNTAYLQM
7615 - QCGGSLRLSCAASGFDIYDDDIHWVRQAPGKGLEWVAYIAPSYGYTDYADSVKGRFTISADTSKNTAYLQM

                                       GGGGS Linker
   EF       90        100            110                10         20
7614 - NSLTAEDTAVYYCSRSSDASYSYSAMDYWGQGTLVTVSSGGGGSDIQLTQSPASLAVSLGQRATISCRASK
7615 - NSLTAEDTAVYYCSRSSDASYSYSAMDYWGQGTLVTVSSGGGGSDIQLTQSPASLAVSLGQRATISCRASK

                     VL1
     30         40         50         60         70         80         90
7614 - SVSTSGYSYMHWYQQKPGQPPKLLIYLASNLESGVPARFSGSGSGTDFTLNIHPVEEEDAATYYCQHSREF
7615 - SVSTSGYSYMHWYQQKPGQPPKLLIYLASNLESGVPARFSGSGSGTDFTLNIHPVEEEDAATYYCQHSREF

    100
7614 - PWTFGGGTKLEIK
7615 - PWTFGGGTKLEIK
```

Supplementary Figure S14 | Sequence alignment of the bispecific diabodies with disulfide bridges, 7614 and 7615. The amino acids are numbered according to the Kabat numbering scheme. Amino acids that share the same residue number are marked with a bar above the sequence.

```
VH1
        10   AB     20        30          40        50          60  C"D
7682 - QVQLQQSGPGLVKPSQTLSLTCAISGDSVSSNSAAWGWIRQSPGRGLEWLGIIQKRSKWYNNYAVKVKSKI
7683 - EVQLVQSGAEVKKPGESLKISCKGSGYSFTNYWVG  WVRQMPGKGLEWMGFIDP SDSYTNYAPKFQGKV
7684 - *VQLQQPGAELVQPGTSVRLSCKASGYIFTTYWIH  WVKQRPGQGLEWIGEINP NNGRINYNEKFKTKA

GGGGS Linker
   70        80   EF    90        100         110                 10
7682 - TINPDTSKNQFSLQLSCVTPEDTAVYYCARYSYPFYSI  DYWGQGTLVTVSSGGGGSQSVLTQPAS*VSG
7683 - TISADKSISTAYLQMSCLTASDTAMYYCARELYQGYMDTFDSWGQGTLVTVSSGGGGSQSVLTQPAS*VSG
7684 - TLTVDKSSSTAYMQLSCLTSEDSAVYYCTRVGVMITTF  PYWGQGTLVTVSSGGGGSQSVLTQPAS*VSG

VL2
       20        30        40        50        60        70        80
7682 - SPGQSITISCAGTSSDVGGYNYVSWYQQHPGKAPKLMIYEDSKRPSGVSNRFSGSKSGNTASLTISGLQAE
7683 - SPGQSITISCAGTSSDVGGYNYVSWYQQHPGKAPKLMIYEDSKRPSGVSNRFSGSKSGNTASLTISGLQAE
7684 - SPGQSITISCAGTSSDVGGYNYVSWYQQHPGKAPKLMIYEDSKRPSGVSNRFSGSKSGNTASLTISGLQAE

Interchain Linker
        90       100
7682 - DEADYYCISYISSNTRLFGGGTKLAVLPWLVPRGSHHHHHHSGGGGSGGGGSGGGGSGGGGLVPRGSPGEV
7683 - DEADYYCISYISSNTRLFGGGTKLAVLPWLVPRGSHHHHHHSGGGGSGGGGSGGGGSGGGGLVPRGSPGEV
7684 - DEADYYCISYISSNTRLFGGGTKLAVLPWLVPRGSHHHHHHSGGGGSGGGGSGGGGSGGGGLVPRGSPGEV

VH2
       10  AB     20        30        40        50        60  C"D   70
7682 - QLVESGGSLVKPGGSLRLSCAASGFTFSNYSMNWVRQAPGKGLEWISSISGSSRYIYYADFVKGRFTISRD
7683 - QLVESGGSLVKPGGSLRLSCAASGFTFSNYSMNWVRQAPGKGLEWISSISGSSRYIYYADFVKGRFTISRD
7684 - QLVESGGSLVKPGGSLRLSCAASGFTFSNYSMNWVRQAPGKGLEWISSISGSSRYIYYADFVKGRFTISRD

GGGGS Linker
       80    EF     90       100        110                   10        20
7682 – NATNSLYLQMNCLTAEDTAVYYCVRGIIDKFDYWGRGTLVTVSAGGGGSDIVLTQPPS*VSVAPGQTARIS
7683 - NATNSLYLQMNCLTAEDTAVYYCVRGIIDKFDYWGRGTLVTVSAGGGGSDIQMTQSPSSLSASVGDRVTIT
7684 - NATNSLYLQMNCLTAEDTAVYYCVRGIIDKFDYWGRGTLVTVSAGGGGSDIQMTQSPASLSASVGETVTIT

VL1
       30        40        50        60        70        80        90
7682 - CSGDNIGSYYVHWYQQKPGQAPVLVIYEDSERPSGIPERFSGSNSGNTATLTISGTQAEDEADYYCSSYDD
7683 - CRASQSIGLYLAWYQQKPGKAPKLLIYAASSLQSGVPSRFSGSGSGTDFTLTISSLQPEDFATYYCQQGNT
7684 - CRASGNIHNYLAWYQQKQGKSPQLLVYNAKTLADGVPSRFSGSESGTQYSLKINSLQPEDFGSYYCQHFWS

       100
7682 - PNFQVFGGGTKLTVK
7683 - LS YTFGQGTKVEIK
7684 - TP FTFGSGTKLELK
```

Supplementary Figure S15 | Sequence alignment of the bispecific diabodies with disulfide bridges, 7682-4. The amino acids are numbered according to the Kabat numbering scheme. Amino acids that share the same residue number are marked with a bar above the sequence.

```
                                         VH1
               10        20        30        40        50        60        70
                      AB                                                C"D
7691 – EVQLVESGGGLVQPGGSLRLSCAASGFNFSSSSIHWVRQAPGKGLEWVASISSSSGSTSYADSVKGRFTIS
7694 – EVQLVESGGGLVQPGGSLRLSCAASGFTFRNSAMHWVRQAPGKGLEWVSSIWYSGSNTYYADSVKGRFTIS
7695 – EVQLVESGGGLVQPGGSLRLSCAASGFTFRNSAMHWVRQAPGKGLEWVSSIWYSGSNTYYADSVKGRFTIS

                                                                 GGGGS
                                                                 Linker
              80        90        100                 110
                      EF
7691 – ADTSKNTAYLQMNSLCAEDTAVYYCARGYYYTGLWYPYAMYEFGMDYWGQGTLVTVSSGGGGSDIQMTQSP
7694 – RDNSKNTLYLQMNSLCAEDTAVYYCARFAGGWGAY          DVWGQGTLVTVSSGGGGSQSVLTQPA
7695 – RDNSKNTLYLQMNSLTACDTAVYYCARFAGGWGAY          DVWGQGTLVTVSSGGGGSQSVLTQPA

                                         VL2
        10       20          30       40       50         60        70
7691 – SSLSASVGDRVTITCRASQ   SIGLYLAWYQQKPGKAPKLLIYAASSLQSGVPSRFSGSGSGTDFTLTIS
7694 – S*VSGSPGQSITISCAGTSSDVGGYNYVSWYQQHPGKAPKLMIYEDSKRPSGVSNRFSGSKSGNTASLTIS
7695 – S*VSGSPGQSITISCAGTSSDVGGYNYVSWYQQHPGKAPKLMIYEDSKRPSGVSNRFSGSKSGNTASLTIS

                                              Interchain Linker
          80        90         100
7691 – SLQPEDFATYYCQQGNTLS YTFGQGTKVEIKPWLVPRGSHHHHHHSGGGGSGGGGSGGGGSGGGGLVPRG
7694 – GLQAEDEADYYCISYISSNTRLFGGGTKLAVLPWLVPRGSHHHHHHSGGGGSGGGGSGGGGSGGGGLVPRG
7695 – GLQAEDEADYYCISYISSNTRLFGGGTKLAVLPWLVPRGSHHHHHHSGGGGSGGGGSGGGGSGGGGLVPRG

                                          VH2
                 10        20        30        40        50        60
                        AB                                             C"D
7691 – SPGEVQLVQSGAEVKKPGESLKISCKGSGYSFTNYWVGWVRQMPGKGLEWMGFIDPSDSYTNYADSVKGRF
7694 – SPGEVQLVESGGSLVKPGGSLRLSCAASGFTFSNYSMNWVRQAPGKGLEWISSISGSSRYIYYADFVKGRF
7695 – SPGEVQLVESGGSLVKPGGSLRLSCAASGFTFSNYSMNWVRQAPGKGLEWISSISGSSRYIYYADFVKGRF

                                                             GGGGS
                                                             Linker
         70        80          90        100          110                10
                         EF
7691 – TISADKSISTAYLQMSSLTACDTAMYYCARELYQGYMDTFDSWGQGTLVTVSSGGGGSDIQMTQSPSSLSA
7694 – TISRDNATNSLYLQMNSLCAEDTAVYYCVRGIIDKF    DYWGRGTLVTVSAGGGGSDIVLTQSPATLSL
7695 – TISRDNATNSLYLQMNSLTACDTAVYYCVRGIIDKF    DYWGRGTLVTVSAGGGGSDIVLTQSPATLSL

                                         VL1
          20        30        40        50        60        70        80
7691 – SVGDRVTITCRASQ SVSSAVAWYQQKPGKAPKLLIYSASSLYSGVPSRFSGSRSGTDFTLTISSLQPEDF
7694 – SPGERATLSCRASQSVSSNYLAWYQQKPGQAPRLLIYDSSSRATGVPARFSGSGSGTDFTLTISSLEPEDF
7695 – SPGERATLSCRASQSVSSNYLAWYQQKPGQAPRLLIYDSSSRATGVPARFSGSGSGTDFTLTISSLEPEDF

          90        100
7691 – ATYYCQQSSSSLITFGQGTKVEIK
7694 – AVYYCHQYSDISPTFGQGTKVEIK
7695 – AVYYCHQYSDISPTFGQGTKVEIK
```

Supplementary Figure S16 | Sequence alignment of the bispecific diabodies with disulfide bridges, 7691, 94 and 95. The amino acids are numbered according to the Kabat numbering scheme. Amino acids that share the same residue number are marked with a bar above the sequence.

Supplementary Table S1. Summary of the Crystallization and Crystal Freezing Conditions

monospecific diabody	crystallization solution	cryoprotectant*
4921	0.1 M Sodium citrate pH 4.5, 0.3 M Ammonium acetate, 30% PEG4000	30% glycerol
4922	0.1 M MES pH 6.5, 2.3 M Sodium acetate, 5% glycerol	
4960	0.1 M Citric acid pH 3.5, 22% PEG10000	30% ethanol
4970	0.1 M Citric acid pH 3.5, 1.0 M Sodium chloride, 30% 2-methyl-2,4-pentanediol	
4971	0.1 M Tris HCl pH 8.5, 0.15 M Magnesium chloride, 32% PEG3350	
6052	0.1 M Sodium citrate pH 4.6, 0.2 M Ammonium sulfate, 18% PEG8000	30% ethylene glycol
**bispecific diabody**	**crystallization solution**	**cryoprotectant***
4937	0.1 M Sodium citrate tribasic pH 5.6, 27.5% PEG12000, 0.1 M Sodium iodide	10% glycerol
6277	0.2 M Ammonium sulfate, 10% PEG 8000	25% ethylene glycol
7128	0.1 M Sodium acetate pH 4.2, 2 M Ammonium sulfate	25% ethylene glycol
6683	0.1 M Sodium citrate tribasic pH 5.5, 19.5% PEG 2,000, 0.75 M Sodium chloride	20% ethylene glycol

* The crystal freezing solutions are composed of the cryoprotectants and the crystallization solutions.

Supplementary Table S2. Summary of the Diabodies

monospecific diabody	antibody sequence database and code number	antigen
4921	PDB, 2V7N	CitS
4922	PDB, 2V7N	CitS
4960	PDB, 2V7N	CitS
4970	PDB, 2V7N	CitS
4971	PDB, 2V7N	CitS
6052	PDB, 2V7N	CitS
**bispecific diabody**	**antibody sequence database and code number**	**antigens**
4937	generated by us, obtained from Dr. Kossiakoff	Proteasome Lid complex, MBP
6277	PDB, 2V7N, 3ULU	CitS, TLR3
7128	generated by us, obtained from Dr. Kossiakoff	Repebody, MBP
6683	generated by us, PDB, 3ULU	Proteasome Lid complex, TLR3

Supplementary Table S3. Crystallographic Data of the Monospecific Diabodies

**Data collection**	4921	4922	4960	4970	4971	6052
Space Group	C2	$P6_122$	$P2_12_12_1$	$P2_12_12_1$	C2	$P2_12_12_1$
Resolution (Å)	2.8 - 50	2.3 - 50	2.0 - 50	1.7 - 50	2.7 – 50	2.0 - 20
Unit cell						
a, b, c (Å)	120.5, 130.0, 38.3	134.1, 134.1 76.3	37.0, 99.6, 128.8	38.0, 114.0, 128.5	164.8, 54.1, 54.7	114.1, 128.8, 259,8
a, β, γ (°)	90, 101.9, 90	90, 90, 120	90, 90, 90	90, 90, 90	90, 103.4, 90	90, 90, 90
R_{sym}	0.135 (0.43)	0.141 (0.54)	0.095 (0.43)	0.051 (0.41)	0.096 (0.55)	0.108 (0.39)
I/σI	10.5 (1.9)	24.2 (4.3)	19.0 (2.5)	34.5 (2.8)	14.8 (1.6)	18.6 (3.7)
Completeness (%)	98.6 (96.5)	99.9 (96.5)	96.3 (91.2)	99.6 (98.8)	98.7 (98.8)	96.6 (95.5)
Redundancy	3.5	17.4	5.6	6.7	3.3	5.1
Search probes	6052*	6052*	6052*	6052*	6052*	2V7N**
**Refinement**						
No. of reflections (work/test set)	16747/837	18570/928	31673/1583	68631/1995	13270/734	247263/1993
R_{work}/R_{free}	0.185/0.259	0.184/0.220	0.164/0.215	0.198/0.231	0.234/0.298	0.259/0.294
No. of atoms						
Protein	3416	1727	3465	3438	3424	23131
Water	99	222	370	507	13	1810
Average B factor ($Å^2$)	56.71	39.07	24.98	32.26	64.54	43.64
r.m.s deviations						
Bond length (Å)	0.008	0.009	0.006	0.006	0.011	0.008
Angles (°)	1.081	1.370	0.904	0.893	1.176	1.299
Ramachandran Plot (%)						
Favored	93.18	98.22	95.80	96.40	95.25	95.50
Allowed	6.82	1.78	4.20	3.60	4.75	4.47
Outliers	0	0	0	0	0	0.03
PDB code	5GRW	5GRV	5GRX	5GRY	5GRZ	5GS1

Values in parentheses represent the highest resolution shell. *diabody name. **PDB code number.

Supplementary Table S4. Crystallographic Data of the Bispecific Diabodies

**Data collection**	4937	6277	6683	7128
Space Group	C2	$P2_1$	$P2_1$	H32
Resolution (Å)	1.95 - 50	1.70 - 50	3.30 - 50	3.60 – 50
Unit cell				
a, b, c (Å)	197.3, 50.8, 105.9	58.0, 78.5, 60.0	98.5, 141.3, 150.8	278.9, 278.9, 132.3
a, β, γ (°)	90, 111.2, 90	90, 89.9, 90	90, 106.8, 90	90, 90, 120
R_{sym}	0.094 (0.43)	0.046 (0.49)	0.076 (0.39)	0.139 (0.48)
$I/\sigma I$	14.2 (2.0)	38.7 (2.5)	18.5 (2.5)	20.7 (4.4)
Completeness (%)	98.9 (94.8)	99.1 (96.9)	96.6 (94.1)	100 (100)
Redundancy	3.2	6.4	3.2	10.2
Search probes	1EZV*, 3PGF*	6052**	4937**, 3ULU*	3PGF*, 3RFJ*, 4J8R*
**Refinement**				
No. of reflections (work/test set)	66332/2000	56429/1984	58301/2795	22880/1999
R_{work}/R_{free}	0.197/0.237	0.192/0.211	0.192/0.248	0.217/0.278
No. of atoms				
Protein	6344	3394	17641	8538
Water oxygens	809	417	0	0
Average B factor ($Å^2$)	24.98	29.52	102.50	92.18
r.m.s deviations				
Bond length (Å)	0.008	0.006	0.011	0.013
Angles (°)	0.970	0.853	1.370	1.681
Ramachandran Plot(%)				
Favored	95.86	97.00	91.89	92.12
Allowed	4.14	3.00	8.07	7.52
Outliers	0	0	0.05	0
PDB code	5GRU	5GS3	5GS0	5GS2

Values in parentheses represent the highest resolution shell. *PDB code number, **diabody name
